# HIV-1 Disease-Influencing Effects Associated with *ZNRD1*, *HCP5* and *HLA-C* Alleles Are Attributable Mainly to Either *HLA-A10* or HLA-B*57 Alleles

**DOI:** 10.1371/journal.pone.0003636

**Published:** 2008-11-04

**Authors:** Gabriel Catano, Hemant Kulkarni, Weijing He, Vincent C. Marconi, Brian K. Agan, Michael Landrum, Stephanie Anderson, Judith Delmar, Vanessa Telles, Li Song, John Castiblanco, Robert A. Clark, Matthew J. Dolan, Sunil K. Ahuja

**Affiliations:** 1 Veterans Administration Research Center for AIDS and HIV-1 Infection, South Texas Veterans Health Care System, San Antonio, Texas, United States of America; 2 Department of Medicine, University of Texas Health Science Center, San Antonio, Texas, United States of America; 3 Infectious Disease Clinical Research Program (IDCRP), Uniformed Services University of the Health Sciences, Bethesda, Maryland, United States of America; 4 Infectious Disease Service, San Antonio Military Medical Center (SAMMC), Ft. Sam Houston, Texas, United States of America; 5 Henry M. Jackson Foundation, Wilford Hall United States Air Force Medical Center, Lackland AFB, Texas, United States of America; 6 Department of Microbiology and Immunology, University of Texas Health Science Center, San Antonio, Texas, United States of America; 7 Department of Biochemistry, University of Texas Health Science Center, San Antonio, Texas, United States of America; Federal University of Sao Paulo, Brazil

## Abstract

A recent genome-wide association study (GWAS) suggested that polymorphisms in or around the genes *HCP5*, *HLA-C* and *ZNRD1* confer restriction against HIV-1 viral replication or disease progression. Here, we also find that these alleles are associated with different aspects of HIV disease, albeit mainly in European Americans. Additionally, we offer that because the GWAS cohort was a subset of HIV-positive individuals, selected based in part on having a low viral load, the observed associations for viral load are magnified compared with those we detect in a large well-characterized prospective natural history cohort of HIV-1-infected persons. We also find that because of linkage disequilibrium (LD) patterns, the dominant viral load- and disease-influencing associations for the *ZNRD1* or *HLA-C* and *HCP5* alleles are apparent mainly when these alleles are present in *HLA-A10*- or HLA-B*57-containing haplotypes, respectively. *ZNRD1* alleles lacking *HLA-A10* did not confer disease protection whereas *ZNRD1-A10* haplotypes did. When examined in isolation, the *HCP5-G* allele associates with a slow disease course and lower viral loads. However, in multivariate models, after partitioning out the protective effects of B*57, the *HCP5-G* allele associates with disease-acceleration and enhanced viral replication; these associations for *HCP5-G* are otherwise obscured because of the very strong LD between this allele and a subset of protective B*57 alleles. Furthermore, *HCP5* and *HLA-C* alleles stratify B*57-containing genotypes into those that associate with either striking disease retardation or progressive disease, providing one explanation for the long-standing conundrum of why some HLA-B*57-carrying individuals are long-term non-progressors, whereas others exhibit progressive disease. Collectively, these data generally underscore the strong dependence of genotype-phenotype relationships upon cohort design, phenotype selection, LD patterns and populations studied. They specifically demonstrate that the influence of *ZNRD1* alleles on disease progression rates are attributable to *HLA-A10*, help clarify the relationship between the *HCP5*, *HLA-C* and HLA-B*57 alleles, and reaffirm a critical role of HLA-B*57 alleles in HIV disease. Furthermore, as the protective B*57-containing genotypes convey striking salutary effects independent of their strong impact on viral control, it is conceivable that T cell-based therapeutic vaccine strategies aimed at reducing viral loads may be inadequate for limiting AIDS progression, raising the potential need for complementary strategies that target viral load-independent determinants of pathogenesis.

## Introduction

An important goal of HIV-1 vaccine research is to identify vaccine-induced immune responses that will predict protection from HIV infection or disease. However, this is proving to be an immensely complex task because it is difficult to predict which immunological response(s) in candidate vaccine-challenged animals or *in vitro* studies will convey a protective effect in humans [1], [2], [3]. This is exemplified by the results of the recent STEP trial, which showed that a HIV-1 vaccine that conferred protection in non-human primates not only failed to confer protection in humans, but may have paradoxically increased risk of transmission [3], [4], [5], [6]. This vaccine failure provides greater urgency for determining the precise repertoire of host genetic factors that influence three parameters: risk of HIV acquisition, extent of initial HIV-1 replication (as reflected by the steady-state viral load), and rate of disease progression. This is because these three parameters also may have utility as endpoints for evaluation of vaccine efficacy [7], [8], [9], [10].

In this quest to identify the correlates of protection that might have relevance for vaccine development, the immunological features linked to alleles of class I Human Leukocyte Antigen (*HLA*) genes, which reside in the Major Histocompatibility Complex (MHC) locus, may be especially informative [11]. This is because genetic association studies have provided incontrovertible evidence demonstrating that *HLA* alleles are critical determinants of HIV-AIDS pathogenesis 11, 12, 13, 14, 15, 16, 17, 18. For example, HLA-B*57 is among the most intensively studied alleles in the HIV-AIDS field as it is among the most protective *HLA* class I alleles. In multiple cohorts of HIV-positive individuals possession of a B*57 allele is associated with a slower rate of disease progression and strong virologic control [11], [12], [14], [15], [17], [19], [20], [21], [22]. Thus, it is conceivable that administration of a therapeutic (HIV disease-retarding) vaccine that elicits immunologic features similar to those associated with disease protective *HLA* class I alleles might have benefit in terms of reducing population-level viral load, and consequently, as modeling studies suggest, the pace of the epidemic [1], [2], [23], [24], [25], [26].

The importance of *HLA* alleles in control of HIV replication is further illustrated by the results of a recent genome wide association study (GWAS) because nearly 50% of the top 50 single nucleotide polymorphisms (SNPs) that associated with virologic control were in or around the MHC locus at 6p21.3 [27]. The selection criteria of subjects for the GWAS were based on a therapy-naïve HIV^+^ individual of European descent achieving a steady-state viral load or having a stable low viral load (<1,000 RNA copies/ml), without knowledge of the date of seroconversion [27]. 486 subjects met this criteria from the 30,000 HIV-positive subjects screened retrospectively [27]. The two SNPs that had the strongest association with virologic control were both near HLA-B*57: the first was a SNP in an endogenous retroviral element designated as *HLA* complex *P5* (*HCP5*), and the second SNP was in the 5′-region of the *HLA-C* gene [27]. Also implicated as major determinants of AIDS progression rates were seven SNPs in and around the genes encoding ring finger protein 39 (*RNF39)* and zinc ribbon domain–containing 1 (*ZNRD1*), which are in close proximity to the *HLA-A* locus [27].

Thus, the main inferences of this GWAS were: (i) *HLA-C* and *ZNRD1* are key independent host determinants associated with control of HIV replication and disease progression, respectively, and (ii) *HCP5* may contribute positively to the well-described protective effects attributed to HLA-B*57, as this allele was found to be in nearly 100% linkage disequilibrium (LD) with the HLA-B*5701 allele. This GWAS clearly represents an advance for the HIV-AIDS field, and the results served as a fulcrum to probe further the importance of the MHC locus in HIV-AIDS pathogenesis. To understand further the role of *HLA* alleles in AIDS pathogenesis we reflected on the following three points.

First, the SNPs in *HCP5* and *HLA-C* accounted for ∼9.6% and ∼6.5% of the variability in steady-state viral load, respectively [27]. Despite these high percentages and the prior observation that viral load is highly predictive of clinical outcome [28], [29], these polymorphisms were not among those identified when disease progression was used as a phenotypic endpoint [27]. For example, the association between the *HLA-C* allele and viral load exceeded the threshold for statistical significance imposed by a GWAS, but the association of this allele for disease progression was not statistically significant [27]. Additionally, the SNP in *HCP5* was not identified in the GWAS screen for disease progression, despite being in nearly 100% LD with the *HLA*-B*5701 allele, which in multiple cohorts has been associated with better virologic control, as well as a slower rate of disease progression [15], [17], [19], [21], [27], [30]. These observations (strong association for the SNPs in *HLA-C* and *HCP5* for viral load, but not for disease progression) suggested that perhaps epidemiological considerations, such as the combined analyses of subjects who achieve steady-state viral load and HIV controllers [31], might have inadvertently led to an enrichment of subjects with genotypes that might confer enhanced restriction of viral replication, and under-representation of individuals who have progressive disease during the early stages of infection. Hence, alleles that might associate with disease acceleration may not be fully represented in the study sample examined, and conceivably, this may obscure genotype-phenotype associations that might be detected within the context of a natural history cohort of HIV-1-infected subjects.

Second, there is a high degree of LD between SNPs in the MHC locus and *HLA* alleles [32], [33]. It thus remained conceivable that the effects attributed to the three SNPs identified in the GWAS (*HCP5*, *HLA-C* and *ZNRD1)* were due, in part, to their LD with *HLA* class I alleles that affect viral load and/or disease course. Third, we and others have shown that the host genetic determinants of HIV-AIDS pathogenesis may be population-specific [34], [35], [36]. Since the GWAS was restricted to subjects of European descent, we considered whether the reported SNPs also influenced other populations.

For these three reasons, we sought to achieve greater clarity regarding the phenotypic effects associated with the SNPs in the three genes identified in the GWAS on steady-state viral load and disease progression rates. We conducted these analyses in a large, well-characterized U.S.-based natural history cohort of HIV-1-positive individuals, whose clinical characteristics have been described before [34], [36], [37], [38], [39]. We evaluated the genotype-phenotype associations attributable to the SNPs in *HCP5*, *HLA-C* and *ZNRD1* before and after accounting for patterns of LD with *HLA* alleles that were in close proximity to these SNPs in the entire cohort, and separately in subjects of European and African descent.

## Results

### LD patterns of SNPs with HLA alleles

**Figure 1:**
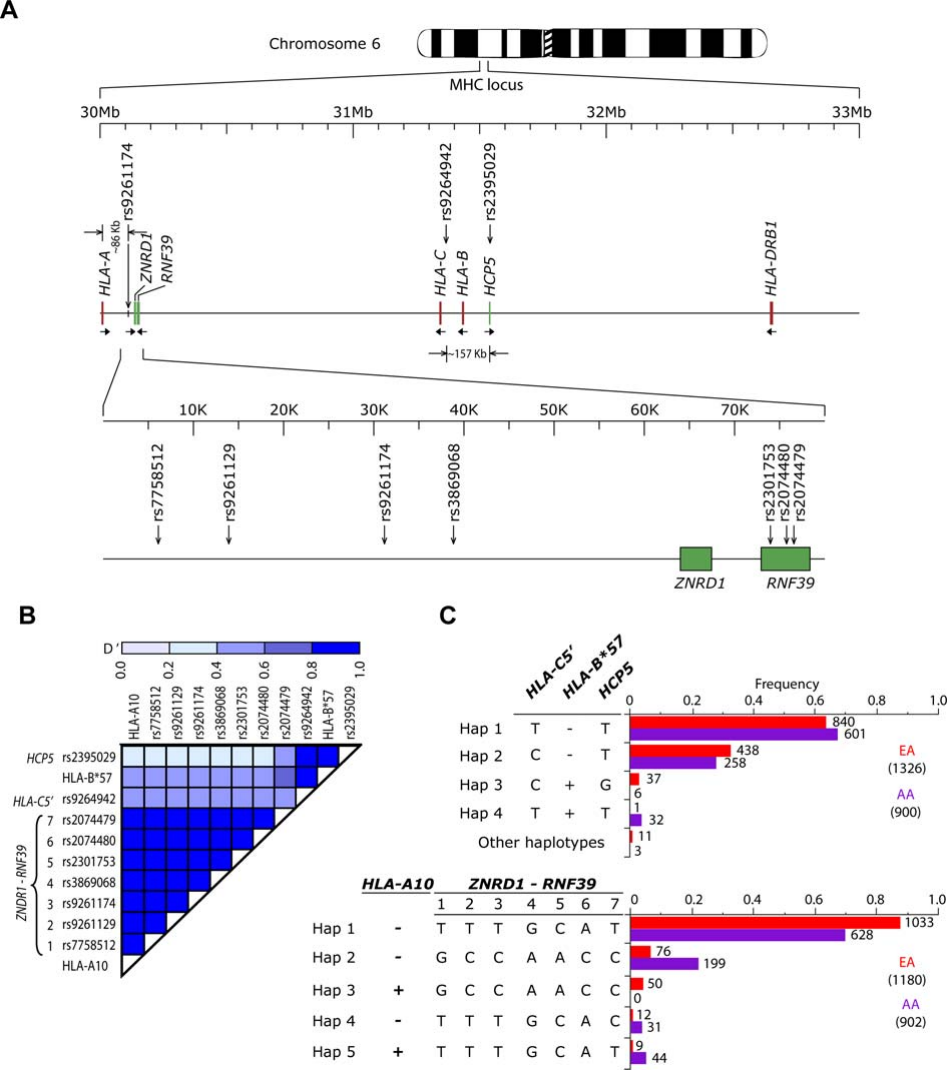
Polymorphisms analyzed in this study and their LD patterns in HIV^+^ subjects from the WHMC cohort. (A) Genomic location of the polymorphisms studied on chromosome 6p21. Green ticks/boxes represent the *HCP5*, *RNF39* and *ZNRD1* genes and red ticks denote *HLA* loci. The relative location of the polymorphisms studied is shown by vertical arrows (along with their respective NCBI rs identifiers). The horizontal arrows under the genes point to the 5′→3′ orientation of the gene. The predicted size of the two extended haplotypes containing *HLA-A10* (∼86kb) and B*57 (∼157kb) are also shown. (B) Linkage disequilibrium (LD) analysis of the studied alleles. D' value estimates are denoted by the intensity of the blue color shown on the scale on the top. (C) Estimated frequencies of the haplotypes based on possession of the HLA-B*57, *HCP5* and *HLA-C5*′ polymorphisms (top), and separately for *HLA-A10* and *ZNRD1* (bottom) polymorphisms in HIV^+^ individuals from the WHMC cohort. Top panel shows the haplotype frequencies for HLA-B*57, HCP5 and *HLA-C5*′ alleles (red and purple colored bars are for EAs and AAs, respectively) while the lower panel is for haplotypes derived based on *HLA-A10* and *ZNRD1* polymorphisms (red and purple colored bars are for EAs and AAs, respectively). Bars represent the haplotype frequency while the numbers to the right of each bar represents the number of haplotypes found in the WHMC subjects. Numbers in parentheses, total number of haplotypes. As discussed in the text, because of the nearly 100% LD between *ZNRD1* and *RNF39* SNPs, rs9261129 was used to represent the *ZNRD1* SNP in statistical analyses (SNP number 3 in panels B and panel C, bottom).

We genotyped HIV^+^ and HIV^−^ subjects in the Wilford Hall Medical Center (WHMC) cohort for the SNP in *HCP5* (rs2395029; designated as *HCP5-T>G*), the 5′-region of the *HLA-C* gene (rs9264942, designated as *HLA-C5*′*-T>C*), as well as for the seven SNPs in or near the *ZNRD1* and *RNF39* genes (Figure 1A). We had previously genotyped subjects from the WHMC HIV^+^ cohort for *HLA* class I alleles [38]. We evaluated the pair-wise patterns of LD between the SNPs, as well as between the SNPs and *HLA* class I alleles in HIV^+^ individuals. The seven SNPs near the *ZNRD1* locus that were found to be associated with inter-subject differences in HIV disease course in the GWAS were all found to be in nearly 100% LD with each other (Figure 1B and Figure 1C, bottom). Predictably, consistent with these very high LD patterns, the genotype-phenotype associations reported below did not differ significantly when we analyzed each of the seven SNPs near *ZNRD1* separately (data not shown). Hence, the results reported here are for a single SNP near *ZNRD1* (rs9261174; third *ZNRD1-RNF39* SNP shown in Figure 1B and Figure 1C, bottom), and this polymorphism is designated here as the *ZNRD1-T>C* SNP.

The spatial proximity between the *HLA-A* locus and the *ZNRD1* SNP (**Figure 1A**) prompted us to investigate whether the latter SNP was in LD with specific *HLA-A* alleles. We considered the results of de Bakker et al [32] who found a high LD between HLA-A*2601 and the SNP designated as rs2301751. The latter SNP is 244 bp from rs2301753, which is one of the seven *ZNRD1-RFN39* SNPs that we genotyped. Furthermore, rs2301753 is reported to be in very high LD (D' = 1.0) with rs2301751 by the HapMap project in Utah residents of European descent. Accordingly, we found a very strong LD between the *ZNRD1* SNP with not only HLA-A*26, but also with A*25, which along with HLA-A*34 and HLA-A*66 make up the serogroup *HLA-A10* [40] (**Figure 1B** and **Figure 1C** **,** bottom). Among the WHMC HIV^+^ subjects, the *HLA-A10* group was composed mainly of HLA-A*25 and HLA-A*26 alleles (89% of the alleles that categorize to the *HLA-A10* serogroup) in European Americans (EAs), whereas HLA-A*34 and HLA-A*66 alleles (78% of the alleles that categorize to the *HLA-A10* serogroup) were more common in African Americans (AA; **Table 1** **in Materials S1**). This difference in the distribution of alleles that belong to the *HLA-A10* serogroup across the two major ethnic groups in the WHMC HIV^+^ cohort was statistically significant (χ^2^ = 72.7; *P* = 1.1×10^−15^).

Consistent with the results of the GWAS [27], a nearly 100% LD was noted between the *HCP5* SNP and B*5701 (**Figure 1B** and **Table 2** **in Materials S1**). Moreover, there was a high degree of LD between the *HCP5-G*, *HLA-C5*′*-C* and HLA-B*57 alleles, and the *HLA-C5*′-*C*/HLA-B*57/HCP5-G-containing haplotype was found almost exclusively in European Americans (**Figure 1B** and **Figure 1C**, top). No significant differences were found in the frequency of these SNPs among HIV-infected and -uninfected individuals (**Table 3** **in Materials S1**).

Because of the observed LD patterns, in the analyses described below, we examined whether the influence on disease course of the SNPs in *ZNRD1* or *HCP5* and *HLA-C* were independent of *HLA-A10* and HLA-B*57, respectively.

### Effects of ZNRD1 SNP on HIV disease

**Figure 2:**
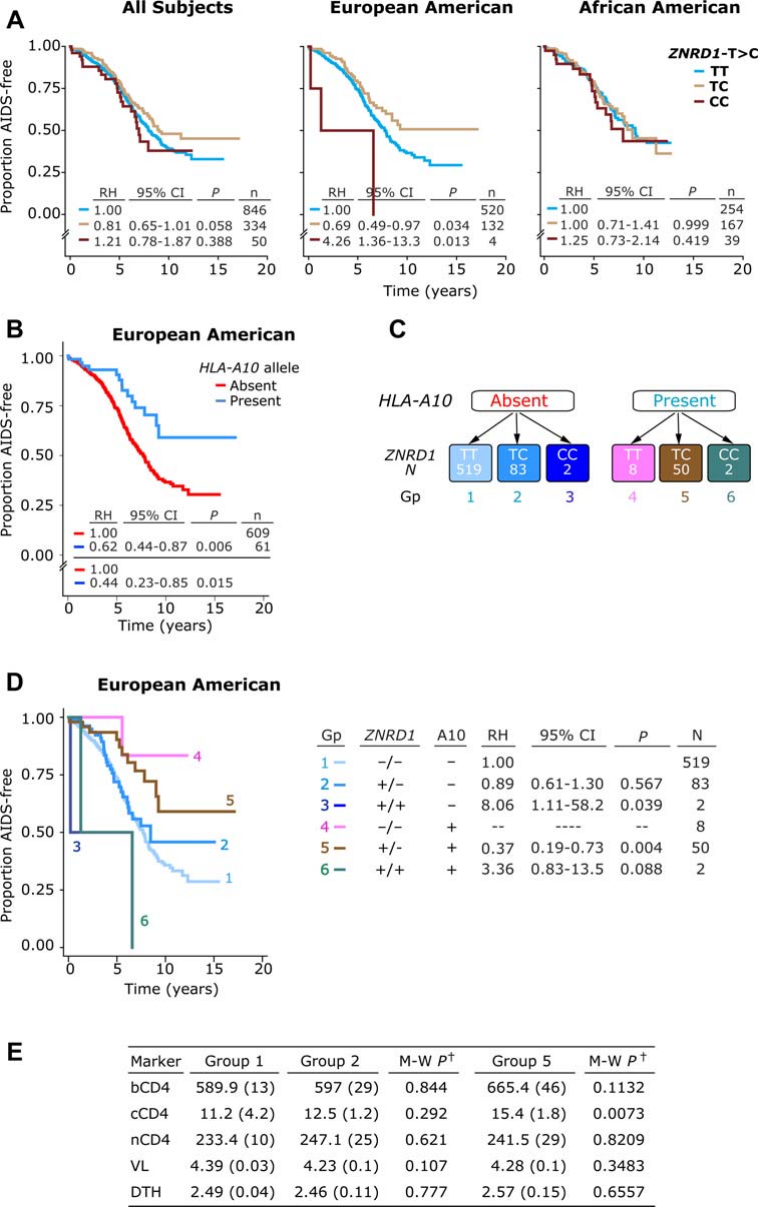
Disease-influencing effects associated with *HLA-A10*/*ZNRD1*-containing genotypes. (A) Plots are Kaplan-Meier curves for time to AIDS (1987 criteria). Disease-influencing effects of the *ZNRD1* SNP in all subjects (left column), and in the European American (middle column) and African American (right column) components of the WHMC cohort. (B) Kaplan-Meier plots depicting the influence on rate of disease progression of alleles categorized into the *HLA-A10* serogroup. The relative hazards (RH) and 95% confidence intervals (CI) were computed before (values above the complete horizontal line) and after (values below the complete horizontal line) adjustment for ethnic background, cohort membership across different therapy eras, *CCL3L1-CCR5* genetic risk group status and whether date of seroconversion was known. (C) Six genotypic groups (enumerated, color-coded) were derived by accounting for *HLA-A10* status and the *ZNRD1* genotype. Number of subjects in each genotypic group are shown within the color-coded boxes. (D) Kaplan-Meier plot for the disease-influencing effects of the six genotypic groups (Gp) shown in panel C. RH, CI, and significance values are depicted to the right and were obtained by Cox proportional hazards modeling. n, number of subjects. Group 1 (Lacking *HLA-A10* and *ZNRD1-C*) is used as a reference. (E) Association between *HLA-A10/ZNRD1*-containing genotypes with virologic-immunologic markers of HIV disease. Group 1 (lacking *HLA-A10* and *ZNRD1-C*) is used as a reference and comparisons are made to *HLA-A10/ZNRD1* genotypic groups 2 and 5. †M-W *P* refers to significance values obtained by Mann-Whitney test. bCD4, nCD4, and cCD4 represent baseline and nadir CD4^+^ T cell counts (cells/mm^3^, mean (±SE)), and cumulative CD4^+^ T cell count (x10^5^ cell-days/mm^3^, mean (±SE)), respectively; the latter parameter provides a measure of the change in CD4^+^ T cell count during disease course as described previously [39]. VL, steady-state viral load (log_10_ HIV mRNA copies/ml, mean (±SE)). DTH, delayed type hypersensitivity skin test reactivity (an in vivo parameter of cell-mediated immunity) was examined as described previously [39] and data reflect the mean (±SE) number of positive skin tests out of the four applied.

In the entire cohort of WHMC HIV^+^ subjects, heterozygosity but not homozygosity for the *ZNRD1-C* allele was associated with disease-retardation (**Figure 2A**, left panel). We stratified the cohort based on whether subjects were EAs or African Americans (AAs), and the stratified analysis revealed that the disease-retarding effects associated with the *ZNRD1-C* allele was restricted mainly to EAs (**Figure 2A** **,** middle and right panel). These results are consistent with the observations by Fellay et al [27] who found that the *ZNRD1-C* allele is associated with a slower disease course in subjects of European descent. However, we also found that this allele does not influence HIV disease course in HIV-positive African Americans in the WHMC cohort.

We next determined whether the disease retarding effects associated with the *ZNRD1-C* allele were due to those associated with alleles categorized to the *HLA-A10* serogroup for three reasons. First, the *ZNRD1-C* allele was in high LD with *HLA-A10* (**Figure 1B** and **Figure 1C**, bottom) and of the 60 HIV-positive EAs who possessed an allele corresponding to the serogroup *A10*, 52 subjects also possessed at least one *ZNRD1-C* allele. Second, the *ZNRD1-C* allele was associated with a reduced rate of disease progression to AIDS only in HIV^+^ EAs (**Figure 2A**). Similarly, only HIV^+^ EAs who possessed alleles corresponding to the *HLA-A10* serogroup had a significantly slower rate of progression to AIDS compared to those who lacked alleles corresponding to the *A10* serogroup (**Figure 2B**). An association between *HLA-A10* and disease course was not detected in HIV^+^ AA (data not shown). Notably, the strength of the disease-retarding effects (relative hazards, RH) associated with *HLA-A10* were similar before (RH = 0.62) and after (RH = 0.44) adjustment for explanatory covariates that in previous studies we had found influenced disease progression in this cohort ([39]; **Figure 2B**). The strength of the protective effects associated with *HLA-A10* were also consistent when the analysis was restricted to subjects who had not received highly active antiretroviral therapy (HAART) (RH = 0.52; 95% confidence interval (CI) = 0.028–0.93; *P* = 0.028 for comparison of those possessing and lacking *HLA-A10*; **Figure S1**). Third, we surmised that if both *HLA-A10* and *ZNRD1-C* each had independent effects on disease, then the protective effects of the *ZNRD1-C* allele that are not because of its LD with *HLA-A10* should be evident in those 85 individuals who possessed at least one *ZNRD1-C* allele but lacked alleles corresponding to *A10* serogroup. For these reasons, we stratified HIV^+^ EAs into six genotypic groups based on their *ZNRD1*-*C* allele and/or *HLA-A10* status (**Figure 2C**). Those lacking both *ZNRD1-C* and *HLA-A10* served as the reference category in the statistical analyses (**Figure 2C**, group 1).

Subjects possessing one *ZNRD1-C* allele but lacking *HLA-A10* (**Figure 2C**, group 2) had a disease course that was indistinguishable from those who lacked both *ZNRD1-C* and *HLA-A10* alleles (group 1). This suggested that in itself the *ZNRD1-C* allele was not associated with disease-retarding effects in the HIV^+^ EAs we examined (**Figure 2D**, compare survival curves for genotypic groups 1 and 2). By contrast, subjects who possessed both a *ZNRD1-C* and a *HLA-10* allele (group 5) had a slower rate of disease progression (**Figure 2D**, compare survival curves for group 5 versus group 1 (*P* = 0.004) or group 2 (*P* = 0.057)). Additionally, although the number of subjects was small, those who were *HLA-A10*+ but lacked the *ZNRD1-C* allele (group 4) also had a slower disease course that was comparable to those in group 5 who were *A10*+ but possessed a *ZNRD1-C* allele (**Figure 2D**). These data indicated that the disease-retarding effect associated with the *ZNRD1-C* allele is evident only when it resides on a haplotype that also contains *HLA-A10*. The failure to detect an effect of the *ZNRD1-C* allele on disease course that is independent of *HLA-A10* did not appear to be due to insufficient sample size because there were more HIV-positive EAs who possessed one *ZNRD1-C* allele but lacked *HLA-A10* (*n* = 83) than those who had one allele each of *ZNRD1-C* and *HLA-A10* (*n* = 50; **Figure 2D**). Furthermore, similar to what was observed for *HLA-A10*, the associations for the six genotypic groups defined by *HLA-A10* and *ZNRD1*-*C* genotypes remained consistent when the analysis was restricted to subjects who had not received HAART (data not shown).

The survival curves associated with homozygosity for *ZNRD1-C* suggested that it might be associated with a rapid rate of disease progression (**Figure 2A**, middle; RH = 4.26; 95% CI = 1.36–13.3; *P* = 0.013). Although there were few subjects who were homozygous for the *ZNRD1-C* allele with (group 6) or without *HLA-A10* (group 3), it was interesting to note that subjects assigned to either of these two groups had a progressive disease course (**Figure 2D**). The small sample sizes preclude firm inferences but the direction of the effects associated with *ZNRD1-A10* genotypic groups 3and 6 suggest that homozygosity for the *ZNRD1-C* allele might associate with disease acceleration even in those instances when these genotypes contain *HLA-A10* alleles.

The results shown in **Figure 2D** were derived from the genotype of *HLA-A10* and *ZNRD1-C* alleles. To confirm that the aforementioned inferences derived from the associations of these compound *HLA-A10/ZNRD1* genotypes are valid, we determined the disease-influencing effects of the individual *A10-ZNRD1* haplotypes shown in **Figure 1C** in the EA component of the WHMC cohort. The results of these analyses conducted at the haplotype level (**Figure S2**) provide further confirmation that the effects associated with the *ZNRD1-C* allele occur only when this allele is present on the same haplotype on which alleles that categorize to the *HLA-A10* serogroup exists. First, haplotype 2 bears the *ZNRD1-C* allele but lacks *HLA-A10* (**Figure 1C**, bottom), and in the heterozygous state this haplotype is not associated with disease retardation and in the homozygous state it is associated with disease acceleration (**Figure S2B**; reference category are those lacking haplotype 2). Second, heterozygosity for the haplotype that bears both *ZNRD1-C* and *HLA-A10* (haplotype 3; **Figure 1C**, bottom) is associated with a slower rate of disease progression (**Figure S2C**; reference category are those lacking haplotype 3). Third, those who are heterozygous for the haplotype that bears *HLA-A10* but not *ZNRD1-C* (haplotype 5; **Figure 1C**, bottom) have a slower disease course than those lacking this haplotype (**Figure S2D**).

Taken together, the analyses conducted thus far at the compound genotype (**Figure 2D**) and haplotype/haplotype pair levels (**Figure S2**) together demonstrate that in the cohort we examined, the *ZNRD1-C* allele does not have independent disease protective effects, and instead, the associations observed for the *ZNRD1-C* allele occur because of its LD with *HLA-A10*, i.e, they are attributable to the disease-modifying effects of alleles corresponding to the *HLA-A10* serogroup. Further underscoring the importance of *HLA-A10* in AIDS pathogenesis in EAs is the observation that the *HLA-A10*-bearing haplotype that lacks *ZNRD1-C*, albeit low in frequency, is associated with disease retardation (**Figure 2D**, group 4, and **Figure S2D**). This suggests further that *HLA-A10* influences AIDS pathogenesis independent of *ZNRD1-C*.

To further confirm that the *ZNRD1*-*C* allele does not influence disease independent of its association with *HLA-A10*, we conducted univariate and multivariate analyses. In univariate analyses, both heterozygosity for *ZNRD1-C* (RH = 0.69; P = 0.030) and *HLA-A10* (RH = 0.46; P = 0.005) associated with a reduced rate of disease progression (**Table 1** under the column designated as ‘Time to AIDS’). However, in multivariate models, *HLA-A10* (RH = 0.51; *P* = 0.028) but not the *ZNRD1*-C (RH = 0.85; *P* = 0.409) allele was associated with a reduced rate of disease progression (**Table 1**).

**Table 1:** HIV disease-influencing effects associated with *HLA-A10* status and *ZNRD1* heterozygosity in European Americans.

Model	Covariates	Time to AIDS			Steady-state viral load			
		RH	95% CI	*P*	Coeff	95% CI	*P*	*R^2^*
1	*ZNRD1* Heterozygosity	0.69	0.49 – 0.96	0.030	−0.14	−0.29 to 0.02	0.077	0.0036
2	*HLA-A10*	0.46	0.27 – 0.79	0.005	−0.07	−0.28 to 0.15	0.534	0.0007
3	*ZNRD1* Heterozygosity	0.85	0.59 – 1.24	0.409	−0.15	−0.33 to 0.03	0.092	
	*HLA-A10*	0.51	0.28 – 0.93	0.028	0.04	−0.21 to 0.29	0.746	0.0055

Results are from multivariate Cox proportional hazards regression (for the outcome of time to AIDS) and linear regression models (for the outcome of log_10_ steady-state viral load).

RH, relative hazards; CI, confidence interval, Coeff, regression coefficient obtained from linear regression model

R^2^, the explained variability in steady-state viral loads was assessed using the R^2^ estimated by analysis of variance (ANOVA). Analysis of *ZNRD1-C* homozygosity was not included in this analysis because of the small sample number.

The aforementioned genotype-phenotype association analyses were conducted using AIDS (1987 criteria) as a phenotypic endpoint. Therefore, it was plausible that although the *ZNRD1-C* allele did not have a direct impact on disease progression rates to AIDS it influenced surrogate markers of disease (e.g., viral load, baseline CD4^+^ T cell count). However, we found that subjects who had one *ZNRD1-C* allele but lacked an *HLA-A10* allele (group 2) were similar to those who lacked both *ZNRD1-C* and *HLA-A10* alleles (group 1) with respect to their baseline, cumulative and nadir CD4^+^ T cell counts, as well as steady-state viral load and delayed type hypersensitivity skin test reactions (a measure of cell mediated immunity [39]) (**Figure 2E**). Subjects in group 5 (heterozygosity for the *HLA-A10/ZNRD1-C*-containing haplotype) were also similar to those in group 1 with respect to most of these laboratory and immunologic characteristics, except those in group 5 had lower cumulative CD4^+^ T cell counts, a parameter that reflects the amount of CD4^+^ T cell loss over disease course and is a parameter that is highly correlated with development of AIDS ([39]; **Figure 2E**). These findings conveyed two points: first, in the cohort examined, the *ZNRD1-C* allele was unlikely to have an impact on disease independent of its LD with *HLA-A10*; and second, the influence of the *HLA-A10/ZNRD1-C*-containing haplotype on disease course occurs independent of an impact on the viral load, and that the influence of this haplotype is restricted mainly to the rate and extent of CD4^+^ T cell loss (assessed by cumulative CD4^+^ T cell count; **Figure 2E**), and by extension, on AIDS progression rates (**Figure 2D**).

To clarify further the relationship between the *ZNRD1-C* allele and steady-state viral load, we determined the influence of *ZNRD1-C* and *HLA-A10* on viral load using univariate and multivariate linear regression analyses. In these analyses, the alleles were considered as independent variables and the steady-state viral load was considered as the dependent variable. These analyses revealed that the impact of the *ZNRD1* allele on viral load was minimal and *HLA-A10* had no influence on viral load (as reflected by the low R^2^ values and non-significant *P* values in **Table 1** under column designated as ‘Steady-state viral load’).

### Effects of HLA-C and HCP5 on disease progression

**Figure 3:**
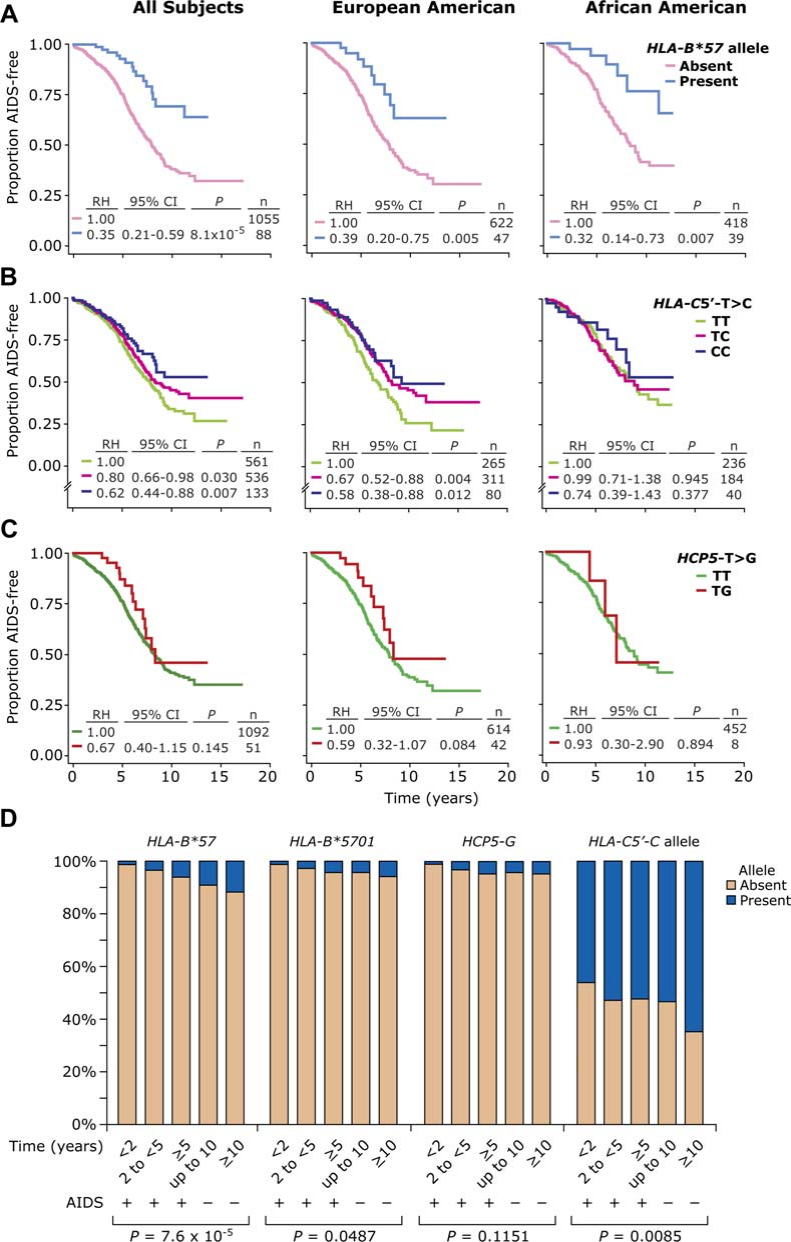
Disease-influencing effects associated with the HLA-B*57, HLA-C5′ and *HCP5* alleles in HIV-infected subjects from the WHMC cohort. (A–C) Plots are Kaplan-Meier curves for time to AIDS (1987 criteria). Disease-influencing effects in all subjects (left column), and in the European American (middle column) and African American (right column) components of the WHMC cohort that are associated with (A) HLA-B*57 alleles, (B) the SNP in *HLA-C5*′ and (C) the SNP in *HCP5*. RH, relative hazards; CI, confidence interval; *P*, significance value obtained by Cox proportional hazards modeling; n, number of subjects. (D) Distribution of the indicated alleles in the WHMC HIV^+^ cohort stratified according to time to AIDS or duration of AIDS-free status. These are indicated as time to AIDS in <2, 2 to <5, and ≥5 years, and no development of AIDS during the first 10 years or after 10 or more years of follow-up. For example, within each panel the left-most bar corresponds to subjects who developed AIDS within 2 years while the right-most bar indicates subjects who did not develop AIDS after 10 or more years of follow-up. At the bottom of each panel is shown significance value (obtained using chi-square test for linear trend) for a linear trend in the proportion of subjects possessing the indicated allele.

**Figure 4:**
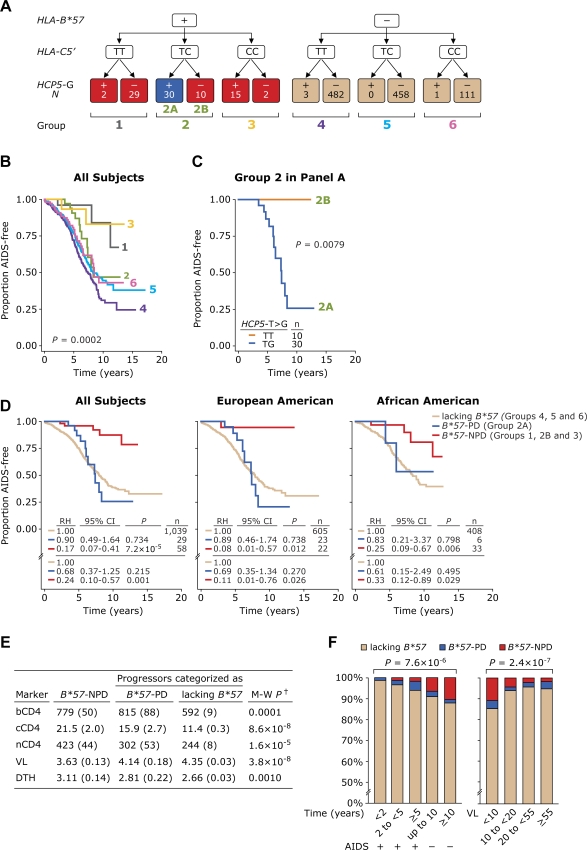
Disease-influencing effects associated with HLA-B*57/HCP5/HLA-C-containing genotypes. (A) Six genotypic groups (enumerated, color-coded) were derived by accounting first for the presence or absence of HLA-B*57 alleles and the wild-type (T/T), heterozygous (T/C) or homozygous (C/C) state for the SNP in *HLA-C.* This group of subjects was stratified further based on presence or absence of the *HCP5-G* allele. Number of subjects in each genotypic group is shown within the color-coded boxes. “+” and “-” denotes “presence” and “absence”, respectively. (B) The disease-influencing effects associated with the six genotypic groups shown in panel A. (C) Kaplan-Meier plots depicting the influence of the *HCP5* SNP on disease progression in subjects in genotypic group 2 stratified according to presence (group 2A) or absence (group 2B) of the *HCP5-G* allele. *P* values in panels B and C are by the logrank test. (D) HLA-B*57/HCP5/HLA-C genotypes that associate with nonprogressive disease (B*57-NPD, i.e., groups 1, 2B and 3 depicted in panels A–C) or progressive disease (B*57-PD; group 2A depicted in panel A and C) compared with genotypes that lack HLA-B*57. RH, relative hazards; CI, confidence interval; *P*, significance value obtained by Cox proportional hazards modeling; n, number of subjects. The RH was computed before (values above complete horizontal line) and after (below) adjustment for ethnic background, cohort membership across different therapy eras, *CCL3L1-CCR5* genetic risk group status and whether date of seroconversion was known. All plots are Kaplan-Meier curves for time to AIDS development (1987 criteria). (E) Association of the genotypes shown in panel D with virologic and immunologic markers of HIV disease. †M-W *P* refers to significance values obtained by Mann-Whitney test for comparison between HLA-B*57-NPD genotype and the remaining cohort subjects. bCD4, nCD4, cCD4, VL and DTH are as described in Figure 2E. (F) The distribution of HLA-B*57-NPD or HLA-B*57-PD genotypes was examined according to time to AIDS or duration of AIDS-free status (left panel) as well as according to steady-state viral load (right panel). Data are from all subjects in the cohort. Time to AIDS or duration of AIDS-free status is shown on the bottom of the left panel and indicates time to AIDS in <2, 2 to <5, and ≥5 years, and no development of AIDS during the first 10 years or after 10 or more years of follow-up. Steady-state viral load shown on the right was categorized as <10,000, 10,000 to <20,000, 20,000 to <55,000 and ≥55,000 copies/ml. *P* value at the top of left panel is significance value for a linear trend obtained using chi-square test for linear trend.

Because of (i) their proximity to HLA-B*57, (ii) the previously reported dominant protective role of B*57 alleles in virologic control and disease progression, and (iii) the high degree of LD between the *HLA-C5*′*-C*, HLA-B*57 and *HCP5-G* alleles (Figure 1B and Figure 1C, top), we determined whether the effects attributable to the SNPs in *HLA-C* and *HCP5* were in part due to these LD patterns and their presence within HLA-B*57-containing genotypes. To this end, we used strategies that were complementary to those we used to clarify the relationship between *ZNRD1* and *HLA-A10* alleles. We first evaluated the associations and distributions separately for the HLA-B*57, *HLA-C5*′ and *HCP5* alleles (**Figure 3**), and then because of their LD patterns, we determined the associations for B*57-containing genotypes that contained or lacked *HLA-C5*′ and *HCP5* alleles (**Figure 4**). We took this analytical approach of first analyzing the genotype-disease-influencing phenotypes at the level of the haplotype and then haplotype pairs (genotypes), as ultimately the phenotypic effect is conveyed by the genotype.

In univariate analyses, possession of a B*57 allele (**Figure 3A**, and **Table 2**, model 1), and heterozygosity and homozygosity for the *HLA-C5*′*-C* allele (**Figure 3B** and **Table 2** **,** model 2) were each associated with significant protective effects on the rate of disease progression to AIDS. The impact of the *HLA-C5*′*-C* allele on disease progression was greater in EAs than AAs (**Figure 3B**). In univariate analyses, the *HCP5-G* allele was associated with protective effects on disease progression, but this trend did not achieve statistical significance and was mostly restricted to EAs (**Figure 3C** and **Table 2** **,** model 3).

**Table 2:** Analysis of the disease-modulating effects of HLA-B*57, the *HLA-C5*′ and *HCP5* alleles/genotypes in HIV^+^ subjects from the WHMC cohort.

Model	Covariates	Time to AIDS			Steady-state viral load			
		RH	95% CI	*P*	Coeff	95% CI	*P*	*R^2^*
1	HLA-B*57	0.38	0.23–0.63	0.0002	−0.53	−0.71 to −0.35	7.1×10^−9^	0.0324
2	*HLA-C5*′							
	Heterozygosity	0.81	0.65–0.99	0.041	−0.07	−0.18 to 0.03	0.172	0.0072
	Homozygosity	0.66	0.46–0.95	0.025	−0.26	−0.42 to −0.09	0.003	
3	*HCP5*	0.67	0.39–1.14	0.135	−0.25	−0.48 to −0.02	0.030	0.0038
4	HLA-B*57	0.39	0.24–0.65	0.0003	−0.51	−0.69 to −0.33	2.9×10^−8^	0.0368
	*HLA-C5*′							
	Heterozygosity	0.81	0.66–1.00	0.051	−0.06	−0.16 to 0.04	0.270	
	Homozygosity	0.72	0.50–1.02	0.067	−0.21	−0.38 to −0.05	0.012	
5	HLA-B*57	0.25	0.13–0.51	0.0001	−0.79	−1.05 to −0.54	1.3×10^−9^	0.0392
	*HCP5*	2.06	0.98–4.33	0.056	0.46	0.14 to 0.78	0.005	
6	HLA-B*57	0.26	0.13–0.51	8.5×10^−5^	-0.82	−1.07 to −0.56	3.7×10^−10^	0.0466
	*HLA-C5*′							
	Heterozygosity	0.81	0.66–0.99	0.043	−0.08	−0.18 to 0.02	0.124	
	Homozygosity	0.69	0.48–0.99	0.044	−0.26	−0.43 to −0.09	0.002	
	*HCP5*	2.19	1.07–4.46	0.032	0.56	0.23 to 0.88	0.001	

Results are from univariate and multivariate Cox proportional hazards regression (for the outcome of time to AIDS (1987 criteria)) and linear regression models (for the outcome of log_10_ steady-state viral load) in all subjects from the WHMC cohort.

RH, relative hazards; CI, confidence interval, Coeff, regression coefficient obtained from linear regression model.

R^2^, the explained variability in steady-state viral loads was measured using the R^2^ estimated by analysis of variance (ANOVA).

Consistent with the results of the univariate analyses, with increasing AIDS-free status, there was a statistically significant step-wise increase in the proportion of subjects possessing a HLA-B*57 allele, including the B*5701 allele (**Figure 3D**). A similar statistically significant trend for a shift in distribution with increasing AIDS-free status was also observed for the *HLA-C5*′*-C* allele (**Figure 3D**). Thus, compared to subjects who developed AIDS within 2 years, HIV^+^ subjects who were AIDS-free for more than 10 years were 12.7, 5.6 and 2.1 times more likely to possess a HLA-B*57, HLA-B*5701 and the *HLA-C5*′*-C* allele, respectively (**Table 3**). Increasing AIDS-free status was also associated with a trend for enrichment of the *HCP5-G* allele although this association was not significant (**Figure 3D** and **Table 3**). It was noteworthy that the degree of enrichment of subjects with the *HCP5-G* allele with increasing AIDS-free status was similar to that observed for the HLA-B*5701 subtype (**Figure 3D**). This observation is consistent with the LD between HLA-B*5701 subtype and the *HCP5-G* allele (D' = 0.91 for EAs and 0.73 for AAs, **Figure 1B**).

**Table 3:** Likelihood of possessing specific alleles in subjects with different rates of HIV disease progression.

Allele	N	Group†	OR (95% CI), *P*	
			Unadjusted	Adjusted‡
HLA-B*57	90	1	1.00	1.00
	145	2	3.18 (0.37–27.66), 0.295	3.10 (0.36–27.04), 0.306
	184	3	5.11 (0.64–40.59), 0.123	4.76 (0.60–37.89), 0.140
	585	4	8.87 (1.21–64.93), 0.032	7.62 (1.02–56.69), 0.047
	168	5	12.71 (1.68–96.16), 0.014	10.80 (1.42–82.05), 0.021
HLA-B*5701	90	1	1.00	1.00
	145	2	2.52 (0.28–22.95), 0.411	2.41 (0.26–22.03), 0.437
	184	3	4.05 (0.50–32.85), 0.191	4.03 (0.49–32.92), 0.194
	585	4	4.14 (0.55–30.89), 0.166	4.42 (0.58–33.81), 0.153
	168	5	5.63 (0.71–44.73), 0.102	5.59 (0.70–44.87), 0.105
*HCP5-G*	98	1	1.00	1.00
	157	2	3.19 (0.37–27.72), 0.293	3.05 (0.35–26.68), 0.314
	198	3	4.62 (0.58–36.99), 0.149	4.63 (0.57–37.35), 0.150
	612	4	4.48 (0.60–33.33), 0.143	4.84 (0.63–36.94), 0.128
	184	5	4.99 (0.62–39.96), 0.130	5.10 (0.63–41.25), 0.127
*HLA-C5*′*-C*	98	1	1.00	1.00
	157	2	1.32 (0.80–2.19), 0.281	1.28 (0.76–2.14), 0.351
	198	3	1.30 (0.80–2.12), 0.285	1.27 (0.77–2.09), 0.341
	612	4	1.36 (0.89–2.09), 0.159	1.47 (0.93–2.32), 0.096
	184	5	2.16 (1.31–3.55), 0.003	2.16 (1.29–3.60), 0.003

Results of multinomial logistic regression analyses and data are derived from all subjects in the HIV+ WHMC cohort.

† Group 1 (progression to AIDS in less than two years) is the reference group, groups 1–5 are as defined in Figure 3D.

‡ Adjusted for seroconversion, race, and the moderate and high *CCL3L1*-*CCR5* genetic risk groups (GRG), which in previous studies were identified as independent determinants of AIDS development [39].

N, number of subjects.

When placed in a multivariate model with HLA-B*57, heterozygosity and homozygosity for the *HLA-C5*′-*C* independently associated with disease-retardation (**Table 2** **,** compare models 2 and 4). However, the statistical significance of the protective effects associated with *HLA-C5*′*-C* in multivariate models were slightly lower than those observed in univariate models (**Table 2** **,** compare models 2 and 4). This was most prominent for homozygosity for *HLA-C5*′*-C* as the hazard ratios for the rate of progression to AIDS were 0.66 (P = 0.025) and 0.72 (P = 0.067) in univariate and multivariate analyses, respectively (compare models 2 and 4, **Table 2**). These results indicated that possibly because of its LD with HLA-B*57, the strength of the disease-retarding effects associated with *HLA-C5*′-*C* may in part be due to the effects conferred by the HLA-B*57 allele.

In univariate analysis, the hazard ratio for the rate of disease progression to AIDS associated with the *HCP5-G* allele was <1 (RH = 0.67, 95% CI = 0.39–1.14, *P* = 0.135), suggesting that this allele might convey weak disease-retarding effects (**Table 2**, model 3). However, when examined in the context of a multivariate model with B*57, the hazard ratio for the *HCP5* allele was >1 (RH = 2.06; 95% CI = 0.98–4.33, *P* = 0.056; **Table 2**, model 5). Conversely, in the univariate analyses, the hazard ratio for rate of disease progression associated with the B*57 allele was 0.38 (*P* = 0.0002, **Table 2** **,** model 1), whereas when examined in the context of a multivariate model with *HCP5-G*, the hazard ratio was lower (RH = 0.25) and the strength of the association increased (*P* = 0.0001 **Table 2** **,** model 5). When *HLA-C5*′*-C* heterozygosity and homozygosity were also added to the multivariate model (**Table 2**, model 6), the strength of the observed detrimental and beneficial effects associated with the *HCP5-G* and B*57 alleles, respectively, on disease progression became even further accentuated (**Table 2** **,** compare models 5 and 6).

We inferred that the opposing direction of the disease-influencing effects associated with the *HCP5-G* allele in the univariate (RH<1, protective effects) and multivariate (RH >1, detrimental effects) analyses, and the accentuation of the protective effects of the B*57 in the multivariate models, convey the following: when examined at the haplotype level, those haplotypes which contain both B*57 and *HCP5-G* alleles are associated with a faster rate of disease progression compared to those B*57-containing haplotypes that bear the *HCP5-T* allele. Consequently, in multivariate models when the overall beneficial effects of B*57 are partitioned out or accounted for, the *HCP5-G* allele, which is in nearly 100% LD with a subset of HLA-B*5*7* alleles, associates with disease-acceleration. Conversely, in multivariate models when the effects of B*57/HCP5-G haplotypes are partitioned out, the overall beneficial effects associated with B*57 are accentuated. Concordant results (and inferences) were obtained when we studied the association of the *HLA-C5*′*–HLA-B–HCP5* haplotypes shown in **Figure 1C** with rates of disease progression (**Figure S3**).

### Influence of HLA-C and HCP5 on viral load

We next examined whether the direction and the relative strength of the effects observed for the HLA-B*57, *HCP5* and *HLA-C5*′ alleles on rates of disease progression are similar to those observed when the steady-state viral load is used as a phenotypic endpoint. The hierarchal order of the extent to which the B*57, *HLA-C* and *HCP5* alleles explained the variability in the steady-state viral load in the WHMC HIV^+^ cohort (R^2^ estimates as percentages are in parenthesis; **Table 2**) was: B*57 (3.2%; model 1)>*HLA-C* (0.72%; model 2)>*HCP5* (0.38%; model 3). These findings indicated that at least within the context of a natural history cohort, the SNPs in *HLA-C* and *HCP5* explained only one-third to one-tenth of the variability in steady-state viral load than the B*57 allele, and several fold less than the reported estimates in the GWAS [27].

To further evaluate the influence of these alleles on viral load, univariate and multivariate linear regression analyses were conducted in which these alleles were considered as independent variables and the steady-state viral load was considered as the dependent variable. Univariate analysis revealed that (i) a HLA-B*57 allele, (ii) one (heterozygosity) or two (homozygosity) copies of the *HLA-C5*′*-C* allele, and (iii) a *HCP5-G* allele each associated with a lower steady-state viral load (as reflected by the negative values of the regression coefficients; **Table 1** **,** models 1 to 3). However, the viral load-mitigating effects observed for possession of the *HCP5-G* allele in the univariate analysis (Coeff = −0.25; *P* = 0.03, **Table 1** **,** model 3) was not evident when this allele was examined in a multivariate context with B*57 alone (Coeff = 0.46; *P* = 0.005, **Table 1** **,** model 5), or with the B*57 allele and *HLA-C5*′ genotypes together (Coeff = 0.56; *P* = 0.001, **Table 1** **,** model 6). Thus, the opposing direction of the viral load-influencing effects of the *HCP5-G* allele observed in the univariate (negative value for the regression coefficients in **Table 1**, model 3; Coeff = −0.25) and multivariate (positive value for the regression coefficients in **Table 1**, models 5 (Coeff = 0.46) and 6 (Coeff = 0.56)) analyses closely mirrored the opposing direction of the disease-influencing effects of the *HCP5-G* allele observed in univariate and multivariate analyses. Thus, once the protective effects associated with HLA-B*57 or *HLA-C5*′*-C* are partitioned out, the *HCP5-G* allele associated with not only a faster rate of disease progression (RH = 2.19; 95% CI = 1.07–4.46; P = 0.032), but also a higher viral load (Coeff = 0.56; 95% CI = 0.23–0.88; *P* = 0.001; **Table 1**, model 6).

Analyses of the final multivariate regression model also revealed that the HLA-B*57, *HLA-C5*′ and *HCP5* alleles together explained 4.66% of the variability in the steady-state viral load in the WHMC HIV^+^ cohort (R^2^ estimate for model 6 in **Table 2**). Additionally, the final model indicates that HLA-B*57 alleles might confer a greater protective effect on early viral replication than *HLA-C5*′ alleles (Coefficient values in model 6 in **Table 2** for B*57 was −0.82, whereas it was *HLA-C5*′ −0.08 and −0.26 for heterozygosity and homozygosity for the *HLA-C5*′*-C* allele).

### Effects of B*57-, HLA-C- and HCP5-containing genotypes

The aforementioned findings indicated that the ‘protective’ phenotype attributable to B*57 (e.g. **Figure 2A**) is a composite, i.e., an overall phenotype that is evident before accounting for the independent phenotypic effects of the SNPs in *HLA-C* and *HCP5-G*. Based on the LD patterns between the HLA-B*57, *HCP5-G* and *HLA-C5*′*-C* alleles (**Figure 1B** and **Figure 1C**, top) and associations described above for each of these alleles, we surmised that a more accurate assessment of the phenotypic effects attributable to B*57 alleles might be attained by evaluation of the genotype-phenotype relationships for B*57-containing genotypes that are defined based on whether or not they also contained the *HCP5-G* and/or *HLA-C5*′*-C* alleles. Conversely, such an approach we surmised would also provide insights into the extent to which the protective effects attributed to the *HLA-C5*′*-C* allele is due to its presence in B*57-containing genotypes. To investigate this, we categorized subjects into six genotypic groups which permitted an analysis of the phenotypic effects associated with *HCP5* and *HLA-C* alleles in genotypes that contained or lacked a B*57 allele (**Figure 4A**).

These six genotypic groups together explained ∼3.1% of variability in steady-state viral load (as analyzed by R^2^) and conferred differential rates of disease progression (**Figure 4B**). Examination of the survival curves for these six genotypic groups revealed the following genotype-phenotype relationships.

Subjects lacking a B*57 allele were assigned to genotypic groups 4 to 6 (**Figure 4A**). Predictably, as group 4 lacked a B*57 allele as well as the protective *HLA-C5*′ allele, subjects within this group displayed the fastest rate of disease progression among the six genotypic groups (**Figure 4B**). It was noteworthy that in those subjects who lacked B*57, but were heterozygous (group 5) or homozygous (group 6) for the *HLA-C5*′*-C* allele had comparable rates of disease progression (**Figure 4B**). This contrasted with the observation that when LD patterns with the B*57 allele had not been accounted for, homozygosity for the *HLA-C5*′*-C* allele was associated with a greater protective effect against disease progression than heterozygosity for this allele (**Figure 3B**). The latter finding indicated that the maximal protective effects of *HLA-C5*′*-C* allele on HIV disease course might be evident when this allele is present in HLA-B*57-containing genotypes, and the data presented below affirm this possibility.

Subjects with a B*57 allele were assigned to genotypic groups 1 to 3, but despite containing a B*57 allele, these genotypic groups were associated with contrasting disease-influencing effects (**Figure 4B**). Compared to subjects assigned to genotypic groups 1 or 3, those in group 2 had a faster rate of disease progression (**Figure 4B**). The distribution of the *HCP5-G* allele in group 2 revealed that individuals assigned to this group could be stratified into two broad categories, with subgroups 2A and 2B possessing or lacking a *HCP5-G* allele, respectively (**Figure 4A**). Remarkably, none of the 10 subjects categorized to group 2B progressed to AIDS (**Figure 4C**), and they all had a genotype that contained a HLA-B*57 and a *HLA-C5*′*-C* allele but lacked a *HCP5-G* allele (**Figure 4A**). By contrast, the *HCP5-G*-carrying subjects categorized to group 2A had a progressive disease (**Figure 4C**). The differential disease outcomes associated with groups 2A and 2B indicated that genotypes which contained a *HCP5-G* allele associated with disease acceleration despite containing two separate ‘protective’ alleles namely a HLA-B*57 and *HLA-C5*′*-C* allele (group 2A). Thus, the genotype-phenotype relationship for group 2A (which contains both HLA-B*57 and *HCP5G* alleles) may explain why when examined in a multivariate model, possession of the *HCP5-G* allele was associated with both disease acceleration and higher viral loads (**Table 2**, model 6).

The phenotype of subjects in group 3 was also very instructive (**Figure 4B**). These subjects were homozygous for *HLA-C5*′*-C* and possessed B*57 and had among the slowest rates of disease progression (**Figure 4B**). This occurred despite the fact that most subjects in group 3 also possessed the *HCP5-G* allele. Thus, among those who possessed B*57, the disease-accelerating effects associated with the *HCP5-G* allele was evident in those with one (group 2A) but not two (group 3) *HLA-C5*′*-C* alleles (**Figure 4B**). These observations underscore our previous thesis [34], [41] that analyses conducted at the allele level obscure complex genotype-phenotype relationships that occur when the phenotypic effects of both haplotypes are accounted for.

These survival curves also underscore two other points: First, a HLA-B*57-containing genotype does not require the presence of a ‘protective’ *HLA-C* allele to convey a protective effect on disease progression. This is reflected by the observation that subjects in group 1 who have a B*57 allele and are homozygous for the *HLA-C5*′*-T* allele have a slow disease course. Second, the maximal ‘protective’ effects associated with possession of a *HLA-C5*′*-C* allele occurs only when it is present in a HLA-B*57-containing genotype. This is reflected by the observation that subjects assigned to B*57-containing genotypic groups 2B or 3 have a much slower disease course than those categorized to B*57-lacking genotypic groups 5 and 6 (**Figure 4** **, B** and **C**, compare survival curves for groups 2B and 3 vs those for groups 5 and 6).

### B*57-containing genotypes and progressive disease

There is clinical, virologic and immunologic data indicating a conundrum: some subjects despite possessing a ‘protective’ B*57 have a progressive disease course [20], [22], [42]. We surmised that the genotype-phenotype relationships shown in **Figure 4B** and **Figure 4C** might address this paradox as they showed that subjects with HLA-B*57-containing genotypes classify into two categories.

The first group are those subjects with genetic features present in genotypic groups 1, 2B or 3 as these genotypic groups are associated with a non-progressive disease (NPD) course (**Figure 4** **, B and C**), and collectively these genotypes are designated here as B*57-NPD genotypes in **Figure 4D**. In the overall cohort, B*57-NPD genotypes conferred a nearly 83% slower rate of disease progression compared to genotypes that lack a B*57 allele (RH = 0.17, 95% CI = 0.07–0.41, *P* = 7.2×10^−5^; **Figure 4D**, left panel). These associations detected at the level of the entire cohort (**Figure 4D**, left panel) remained consistent after adjustment for covariates known to influence disease progression in this cohort, including ethnicity [39], [43].

The second group are those subjects categorized to group 2A and, compared to those with B*57-NPD genotypes, the clinical course of subjects in this group is characterized by a progressive disease (PD; designated as B*57-PD genotype in **Figure 4D**). Remarkably, the disease course of those with a B*57-PD genotype is similar to those who lack a B*57 allele (**Figure 4D**). Consistent genotype-phenotype relationships were also evident when similar analyses were conducted separately in HIV^+^ EAs and AAs before and after adjusting for covariates (**Figure 4D** **;** middle and right panels) or when the analyses were restricted to subjects who did not receive HAART (**Figure S4**). In these HAART-free subjects, those assigned to the B*57-NPD genotypic group had a nearly 80% lower risk of progressing to AIDS than those who lacked a B*57 allele (RH = 0.21; 95% CI = 0.08–0.57; *P* = 0.002).

Based on the aforementioned findings we surmised that the comparison of the immunologic (CD4^+^ T cell profiles and DTH skin test reactivity) and virologic (viral load) profiles would reveal a step-wise worsening in subjects categorized to B*57-NPD, B*57-PD and those lacking a B*57 allele. In general agreement with this, as a general rule, there was a step-wise decrease in the baseline, cumulative and nadir CD4^+^ T cell counts as well as DTH responses in subjects assigned to B*57-NPD, B*57-PD and those lacking B*57 (**Figure 4E**). Conversely, in subjects categorized to B*57-NPD, B*57-PD and those lacking B*57 there was a step-wise increase in the steady-state viral load (**Figure 4E**). The only exception to this general rule was that the baseline CD4^+^ T cell counts in subjects assigned to B*57-NPD and B*57-PD were similar, but they were higher than the cell counts found in those who lacked a B*57 allele (**Figure 4E**).

To validate that B*57-containing NPD and PD genotypes convey contrasting phenotypic effects during HIV disease course we used a complementary approach and determined the distributions of these genotypes as a function of increasing AIDS-free status and steady-state viral loads with the intent of testing two premises. First, although increasing AIDS-free status is associated with a stepwise enrichment of subjects with a B*57-containing genotype (**Figure 3D**), the greatest enrichment should be for those with B*57-NPD genotypes. Second, the B*57-NPD and not B*57-PD genotypes should be enriched for subjects with a low steady-state viral load. The data shown in **Figure 4F** (left panel) provides evidence in support for the first premise. Nearly 12 percent of those HIV-positive individuals who were AIDS-free after 10 years of follow-up possessed a B*57-containing genotype, and strikingly ∼90% of these subjects were those with a B*57-NPD genotype (**Figure 4F**, left panel). The data shown in **Figure 4F** (right panel) provides evidence in support of the second premise as among subjects with a steady-state viral load lower than 10,000 copies/ml, there was an overrepresentation of those with B*57-NPD genotypes. It was noteworthy that the proportion of subjects with a B*57-PD genotype was similar in those who had a steady-state viral load of <10,000 and ≥55,000 copies/ml (**Figure 4F**). Thus, these distribution patterns (**Figure 4F**) provide additional validity for the categorization of B*57-containing genotypes into B*57-NPD and B*57-PD genotypes as a means to improve the accuracy of identifying those B*57-carrying subjects who may have a slower disease course or control of viral replication during the early phases of infection.

### Viral load-independent effects of B*57-NPD genotypes

Based on the well-established, strong relationship between steady-state viral load and AIDS development [28], [29], one interpretation of the aforementioned findings might be that the B*57-NPD genotypes convey disease-retardation by simply influencing the extent of early viral replication. However, in a recent study, we found that although the steady-state viral load is a strong predictor of AIDS risk, it accounted for only ∼12% of the variability in AIDS progression rates [39]. The findings by Rodriguez et al also suggest that the contribution of viral load to the explained variability in the rate of decline in CD4^+^ T cell counts might not be very large [44], [45]. These data indicated that parameters that are independent of viral load are also critical determinants of AIDS pathogenesis. We therefore used nested multivariate Cox proportional hazards models (**Table 4**) to assess whether HLA-B*57-NPD genotypes affected disease progression independent of the viral load.

**Table 4:** Viral load-independent disease-influencing effects of HLA-B*57-containing genotypes.

Model #	Covariates	*N*	B*57-PD RH (95% CI), *P*	B*57-NPD RH (95% CI), *P*
1	None	1,126	0.90 (0.49–1.64), 0.734	0.17 (0.07–0.41), <0.001
2	Baseline CD4 (C)	974	1.33 (0.73–2.44), 0.347	0.22 (0.09–0.54), 0.001
3	Nadir CD4 (N)	974	0.95 (0.52–1.73), 0.867	0.24 (0.10–0.59), 0.002
4	Steady-state VL (V)	977	1.34 (0.73–2.45), 0.342	0.21 (0.07–0.67), 0.008
5	DTH (D)	973	0.66 (0.36–1.22), 0.185	0.20 (0.08–0.48), <0.001
6	Moderate-High GRG (G)	1,102	0.81 (0.44–1.48), 0.492	0.18 (0.07–0.43), <0.001
7	C, N	974	1.17 (0.64–2.14), 0.612	0.27 (0.11–0.66), 0.004
8	C, N, V	829	1.19 (0.65–2.18), 0.582	0.25 (0.08–0.78), 0.017
9	C, N, V, D	828	0.92 (0.50–1.73), 0.806	0.24 (0.08–0.76), 0.015
10	C, N, V, D, G	815	0.85 (0.45–1.62), 0.629	0.25 (0.08–0.80), 0.019

Results are from nested multivariate Cox proportional hazards models for the outcome of time to AIDS (1987 criteria) for subjects assigned to B^*^57-PD and B^*^57-NPD before (none, model 1) and after adjustment for covariates individually (models 2 to 6) or in unison (models 7 to 10). Data is presented as relative hazard (RH) with 95% CI and *P* values. The reference group for all the comparisons is those who lack a B^*^57 allele (relative hazard = 1). N, number of subjects. B^*^57-PD represents subjects who possess B^*^57-containing genotypes that associate with progressive disease whereas B^*^57-NPD represents subjects who possess B^*^57-containing genotypes that associate with non-progressive disease (as shown in **Figure 4D**).

Before accounting for any of the covariates, those HIV-positive persons categorized to B*57-NPD had an ∼83% lower risk of progressing to AIDS compared to individuals who lacked a HLA-B*57 allele (RH = 0.17; 95% CI = 0.07–0.41; **Table 4**, model 1). We next adjusted–individually and in unison–for the disease-influencing effects of several explanatory variables. These analyses revealed that even after adjustment for the disease-influencing effects of these covariates, the B*57-NPD genotypes remained strong independent predictors of a slow rate of disease progression (**Table 4**, models 2 to 10). By contrast, the rates of disease progression among those categorized to B*57-PD and those lacking a B*57 allele were not statistically different (**Table 4**, model 1), and this association remained after inclusion of the other covariates in the model (**Table 4**, models 2 to 10).

## Discussion

The salient genetic-epidemiologic findings of this study conducted in a natural history cohort of HIV-1 subjects are as follows. First, because of their LD patterns, the associations for *ZNRD1* alleles were evident only when these alleles were also present with alleles that categorize to the *HLA-A10* serogroup. Second, heterozygosity and homozygosity for the *HLA-C5*′ allele were associated with disease retardation, but because of LD between *HLA-C* and B*57 alleles, the maximal beneficial effects associated with *HLA-C*-bearing genotypes were evident when they were present in HLA-B*57-containing genotypes. Third, because the *HCP5-G* allele is in nearly 100% LD with the B*5701 allele, the genotype-phenotype relationships observed for genotypes that contain B*57 and *HCP5-G* alleles reflected those of B*5701-containing genotypes. We find that those *HCP5-G*-carrrying individuals who have one copy of *HLA-C5*′*-C* (i.e., heterozygous for *HLA-C5*′*-C*) had a progressive disease course whereas most of those who bear two copies of the *HLA-C5*′*-C* allele (i.e., homozygous for *HLA-C5*′*-C*) have a nonprogressive disease course. Fourth, after partitioning out the protective effects of B*57, the *HCP5-G* allele was associated with disease-acceleration and enhanced viral replication, and these associations for *HCP5-G* were otherwise masked because of its LD with the protective B*57 alleles. Fifth, *ZNRD1* alleles influenced disease course without impacting on the viral load. Sixth, the influence of the *HLA-C* on the steady-state viral load was much lower than that of HLA-B*57 alleles, and the extent of variability in steady-state viral load that can be explained by *HLA-C* alleles independent of HLA-B*57 was significantly lower than that reported previously [27]. Seventh, because of the aforementioned LD and genotype-phenotype patterns, we find that B*57-containing genotypes could be stratified into two categories, one of which associated with striking disease retardation, the other with a disease course that is similar to that observed in individuals who lack a B*57 allele. Finally, we found that B*57-containing genotypes that associate with a slower disease course influenced progression rates by impacting both the viral load as well as parameters that are independent of the viral load. Below, we discuss the seven major implications of these findings within the context of how they apply to AIDS pathogenesis, vaccine development, and genotype-phenotype association studies.

First, our results provide a basis to clarify the role of *ZNRD1* in HIV pathogenesis. Using several different analytical approaches we find that in the European American component of the HIV^+^ cohort we studied, associations of the alleles in or around *ZNRD1* or *RNF39* with disease progression are not attributable to an independent effect of these alleles on HIV disease course, but rather to their strong LD with *HLA-A10* alleles. In the cohort studied, there was a higher proportion of subjects who had *ZNRD1*-containing haplotypes that lacked *HLA-A10* than individuals with *ZNRD1-C/A10-*containing haplotypes. Despite this, *ZNRD1-C/A10-*lacking haplotypes were not associated with a slower rate of disease progression whereas the *ZNRD1-C/A10-*containing haplotypes were. Additionally, *HLA-A10*-containing genotypes that lacked *ZNRD1* alleles were also associated with a slower disease course. Collectively, these findings suggest that *HLA-A10* may be an important determinant of the rate of HIV disease progression in European Americans. Previous association studies also suggest that serogroup *HLA-A10* may convey a protective effect [46]. Additionally, HLA-A*25 (a subtype of *A10*) is known to present gag epitopes [47], [48], [49], and HLA-A*26 (a subtype of *A10*) restricted gag responses have been also reported [50]. These prior reports in conjunction with our genetic epidemiologic findings invoke the possibility that *HLA-A10* carriers elicit protective immune responses against key HIV-1-derived peptides, and consideration of this possibility might have value for understanding the host factors that influence AIDS pathogenesis and for vaccine development. Nevertheless, future studies are clearly warranted to clarify the role of *ZNRD1* in AIDS pathogenesis as it was among the more than 250 candidate genes identified by a large-scale siRNA screen used to identify host factors required by HIV-1 [51].

Second, our results may provide a basis to clarify the disease- or viral load-influencing effects of the SNPs in *HCP5* and *HLA-C*. (i) In multivariate models, after accounting for the protective effects of HLA-B*57 alleles, we find that the *HCP5-G* appears to have detrimental effects on both viral load and disease progression. Thus, we surmise that when examined in isolation the true phenotypic effects associated with the *HCP5-G* allele might be obscured because of its strong LD with protective B*57 alleles. We propose that the protective associations detected previously for the *HCP5-G* allele [27] are perhaps due to a specific set of B*57-containing genotypes, i.e., those that are also homozygous for the *HLA-C5*′*-C* allele (**Figure 4**, group 3). (ii) Based on the LD between B*57 and *HLA-C5*′*-C* alleles and the significant beneficial influence of HLA-B*57-containing genotypes on viral restriction and disease course observed here and in many prior studies [11], [12], [14], [17], [19], [20], [21], [22], we suggest that the dominant protective effects attributable to *HLA-C5*′*-C* alleles may be because of their presence in specific B*57-containing genotypes. (iii) We suggest that the differences in the extent to which the *HLA-C5*′ and *HCP5* alleles explained the variability in the steady-state viral load detected in a natural history cohort versus that observed in the GWAS might relate to epidemiological considerations. The combined analysis of subjects who achieved a steady-state viral load and those who had virologic characteristics similar to those of spontaneous HIV controllers [31], [52], [53] might be one reason why such a strong association for viral load was detected for *HLA-C5*′ and *HCP5* alleles, yet these alleles were not among those detected when using disease progression as a phenotypic endpoint [27]. Consistent with this possibility, the preliminary results of a separate GWAS in HIV controllers also revealed an association between *HLA-C5*′ and *HCP5* alleles and restriction of viral replication [54]. We are mindful that other epidemiologic considerations might also contribute to differing genotype-phenotype associations. This could include the criteria used to define disease progression, which was rate of CD4 decline or time to initiation of HAART in the GWAS [27] versus a clinical endpoint used herein. Thus, it is conceivable that the varying (i) characteristics (e.g., ethnicity, risk behavior) of the subjects, (ii) selection criteria used for entry into the study as well as (iii) phenotypic endpoints used may collectively account for some of the differences in the results of the genotype-phenotype association studies.

Third, the LD patterns detected herein are not completely unanticipated because the MHC locus is known to have small recombination rates and a high degree of LD, hence, increasing the number of extended haplotypic blocks in this genomic area [32]. The nature of these extended haplotypes differ in their genetic composition and the prevalence of these extended haplotypes also vary significantly according to continent-of-origin [32]. Notably, several extended *HLA* haplotypes that have relevance to HIV-AIDS have been identified [55], [56]. LD with protective *HLA* alleles might provide a basis for why a large proportion of the SNPs identified in the GWAS were in the MHC locus, including additional *HCP5* and *HLA-C* SNPs [27]. Similar considerations of LD might also have applicability to the inferences of GWAS for other diseases in which HLA-B*57 and other *HLA* alleles play pathogenic roles. For example, a recent GWAS implicated the *HCP5-G* allele as a determinant of psoriasis [57].

Fourth, we provide a genetic basis for the long-standing but highly underappreciated conundrum of why some B*57-carrying individuals have a progressive disease course whereas others exhibit a strikingly slow clinical course [19], [20], [22], [42]. The prevailing viewpoint is that possession of a B*57 allele confers strong protection against early viral replication and disease progression. However, the studies especially of Migueles et al [19] and Navis et al [22] suggest that a substantial proportion of HIV^+^ subjects who possess a B*57 allele, including those possessing B*5701 can have a progressive disease course. A very striking example of this conundrum was reported recently by Bailey et al who studied a HIV-1 transmission pair [42]: while both transmission pairs were HLA-B*57 positive, the transmitter progressed to AIDS, whereas the recipient was an elite controller. The contrasting clinical phenotypes of nonprogressive vs progressive disease course associated with carriage of a HLA-B*57 allele has been attributed to differences in cytotoxic T lymphocyte (CTL) escape mutations and CTL activity against epitopes in Gag as well as to differential viral replication capacities [20], [22]. Here, we show that specific *HLA-C5*′/HLA-B*57/HCP5 containing genotypes may represent multiplex ‘genetic signatures’ that have differential effects on HIV disease course. These genetic findings have broad biologic and public health significance as the immunologic features linked to protective vs non-protective B*57-containing genotypes may have practical value in achieving a detailed understanding of the immunologic correlates of virologic control and nonprogressive disease.

Fifth, our findings place a spotlight on that aspect of pathogenesis that is influenced by parameters whose effects are independent of the viral burden. This point is illustrated by two observations (i) *HLA-A10* affects disease course without influencing the viral load, and (ii) HLA-B*57-containing genotypes that convey a nonprogressive disease course do so partly by restricting viral replication (as reflected by the viral load) and also by impacting on parameters that are independent of the viral load. These observations are reminiscent of our previous results where we found that *CCL3L1-CCR5* genotypes also influence disease progression, in part, by impacting on parameters that are independent of the viral load [37], [38]. Although the full nature of these viral load-independent parameters is as yet unknown, their importance in pathogenesis is inferred from (i) prior findings showing that the viral load does not explain the full extent of variability in AIDS progression rates [39] or rate of CD4 cell decline [44], [45], and (ii) findings in non-human primates who are naturally infected with simian immunodeficiency virus, as these animals exhibit a non-progressive disease despite high viral loads [58]. Hence, consideration of both the viral load and viral load-independent parameters might help mitigate the confounding that may occur in HIV vaccine and genetic-epidemiologic studies in which the viral load is used as the primary surrogate marker for vaccine efficacy or disease outcome, respectively. Additionally, findings from animal studies [2] and mathematical modeling [23], [24], [25], [26] support the hope that imperfect, T-cell based disease-modifying, i.e., therapeutic vaccines, by reducing plasma viral load at the population level might abate the epidemic. However, the clinical [44], [45], and genotype-phenotype relationships observed herein and previously [39] raise the possibility that for a therapeutic vaccine to be efficacious in mitigating disease progression rates, in addition to reducing the viral load, the vaccine may also need to target pathogenic viral load-independent factors.

Sixth, within the context of a natural history cohort of HIV-positive individuals we find that there is a gradual enrichment of protective HLA-B*57 genotypes in subjects who remain AIDS free for a prolonged duration. This gradual shift emphasizes that the relative contribution of a genetic factor that confers the phenotype of nonprogressive disease within the context of a natural history cohort may not be identical to the contribution of the same genetic factor in subjects who are selected for analyses based on specific clinical characteristics such as those with nonprogessive disease (e.g., long-term nonprogressors), or virologic characteristics such as untreated subjects who maintain very low viral loads during some period of the disease course (e.g., elite or viremic controllers) [31]. For example, Migueles et al [19] found that the HLA-B*5701 allele is dramatically overrepresented in long-term nonprogressors. When studied within the context of a natural HIV history cohort, we find that 6.6% of EAs possess a B*5701 allele and of these 64% have progressive disease. Thus, the very high frequency of the HLA-B*5701 allele among those selected because of nonprogressive disease or a low viral load may represent an estimate specific to this selected subset of HIV-positive subjects, because HIV-positive individuals who have progressive disease are unlikely to be well represented in such a study group. Hence, association studies in such selected patients are more likely to identify genetic factors that are skewed towards having a significant impact on restriction of viral replication or nonprogressive disease, and may not necessarily identify the full realm of host factors that influence pathogenesis in the context of the natural clinical course of HIV disease, especially because a proportion of AIDS pathogenesis is independent of the viral load [39], [44], [45].

Finally, the results of the present study support our previous thesis that the host determinants of HIV pathogenesis are likely to be highly population-specific [34], [36]. Similar inferences were also reported by Winkler and colleagues [35]. In this study, we found that the impact of the *A10/ZNRD1*, *HLA-C* and *HCP5* alleles on disease course were evident mainly in subjects of European descent. Some of these race-specific effects are not related to the differential distribution in the frequency of these polymorphisms in European and African Americans. The exact basis for these race/ethnicity-specific effects on HIV disease is unclear. Given the differing evolutionary histories of the populations examined, one possibility is that the race/ethnicity-specific effects observed might relate to the interplay between polymorphisms in different gene systems that played an ancestral role in the contrasting host-microbe interplay found in subjects of European and African descent. For example, we found recently that the disease-accelerating effects of the *CCL5* -471A/A genotype in African Americans is evident only in those who do not bear the African-specific *-46C/C* genotype of Duffy Antigen Receptor for Chemokines (*DARC*), a genotype which is thought to have arisen due to the selective pressure of specific malaria-causing species [43]. Regardless of the precise reasons for the observed race-specific effects, they add a tier of underappreciated complexity as they point to population-specific correlates of protection. Consequently, the development and evaluation of an effective HIV vaccine might need to factor in not just HIV diversity, but also host genetic diversity.

## Methods

### Study subjects

HIV-positive subjects were from the Department of Defense (DoD) HIV Natural History Study (NHS) cohort followed at Wilford Hall Medical Center (WHMC) and more recently at the Brooke Army Medical Center (BAMC), San Antonio, TX. The studied population is the local component of a prospective multisite observational cohort from the United States Military's Tri-Service AIDS Clinical Consortium (TACC) HIV Natural History Study. Unidentified cast-off blood from subjects participating in training at Lackland AFB, TX was used for the HIV-negative control population. The demographic and clinical characteristics of the HIV-positive WHMC cohort have been described extensively [34], [36], [37], [38], [39], [59], [60], [61]. The voluntary, fully written informed consent of the subjects studied in this research was obtained as required by Air Force Regulation 169-9 and additional approval from the Institutional Review Board (IRB) of the University of Texas Health Science Center, San Antonio, TX.

### Genotyping

Genotyping for HLA-B*57 and HLA-B*5701 subtypes was undertaken in 1,143 HIV^+^ subjects. Polymorphisms in the *HCP5* (rs2395029), *HLA-C5*′ (rs9264942) and seven SNPs in and around *ZNRD1*-*RNF39* genes (**Figure 1A**) were genotyped in 1,230 HIV^+^ subjects and 1,129 HIV-negative subjects. Haplotypes based on *HLA-A10* status and the seven *ZNRD1* SNPs as well those based on HLA-B*57, *HCP5* and *HLA-C5*′ alleles were generated using unphased data. For this purpose, we used the PHASE software. Detailed materials, methods and description of the study cohort are available as supporting online material (Materials S1).

## Supporting Information

Materials S1 (0.20 MB DOC)

Figure S1 Disease-influencing effects associated with HLA-A10 status in HIV-positive EA subjects from the WHMC cohort who had not received HAART.
(0.26 MB TIF)

Figure S2 Association between HLA-A10-ZNRD1 haplotypes and rates of HIV disease progression in the EA component of the WHMC cohort.
(0.58 MB TIF)

Figure S3 Association between HLA-C5′-HLA-B-HCP5 haplotypes and rates of HIV disease progression in the WHMC cohort.
(1.06 MB TIF)

Figure S4 Disease-influencing effects associated with HLA-B*57-NPD and HLA-B*57-PD genotypes in subjects who had not received HAART.
(0.33 MB TIF)
