# Supplementary material for: HIV-1 Disease-Influencing Effects Associated with ZNRD1, HCP5 and HLA-C Alleles Are Attributable Mainly to Either HLA-A10 or HLA-B*57 Alleles
Source: PLoS One. 2008 Nov 4;3(11):e3636. doi: 10.1371/journal.pone.0003636 (PMC2574440; doi:10.1371/journal.pone.0003636)
Supplement: Materials S1 — (0.20 MB DOC) [file pone.0003636.s001.doc]

**HIV-1 Disease-influencing Effects Associated with *ZNRD1*, *HCP5* and *HLA-C* alleles are Attributable Mainly to either *HLA-A10* or *HLA-B*57* alleles**

**Genotyping and nomenclature of SNPs**

Single nucleotide polymorphisms (SNP) in the coding region of *HCP5* (rs2395029 T>G; designated here as *HCP5-T>G*), a SNP that is ~35kb upstream of *HLA-C* (rs9264942 T>C; designated here as *HLA-C5’-T>C*) as well as seven SNPs in and near *RNF39* and *ZNRD1* (rs9261174, rs3869068, rs2074480, rs7758512, rs9261129, rs2301753, and rs2074479) were genotyped using TaqMan based allelic discrimination assays following the manufacturer’s instruction and use of a 7900HT sequence detection system (Applied Biosystems, Foster City, CA). Primers and probes are described below. The SNPs were in Hardy-Weinberg equilibrium in HIV-positive and HIV-negative subjects as well as in the two major ethnic groups represented in the WHMC cohort (Table S3).

| SNP | Primers/Probes | Oligonucleotide sequences |
| --- | --- | --- |
| *HCP5*-T>G  (rs2395029) | Sense primer | 5’-TCTCACCCGCTGGTCTCT-3’ |
|  | Antisense primer | 5’-CAGGGTAGAAGGTCCTGGATTCT-3’ |
|  | Wt Probe | Vic-ACATTACAGCTGCCaCAGG-MGBNFQ |
|  | Mut Probe | 6FAM-CATTACAGCTGCCcCAGG-MGBNFQ |
| *HLA-C5’*-T>C  (rs9264942) | Assay by Demand Assay (C_29901957_10) were ordered from Applied Biosystems (ABI, www.appliedbiosystem.com), primer/probe sequences are patented by ABI | |
| *ZNRD1*_SNP1-T>C  (rs9261174) | Sense primer | 5’-GCCAATACCTTGCTTGCCATTTT-3’ |
|  | Antisense primer | 5’-GCTGGAAGTATCACAGTACCTGACT-3’ |
|  | Wt Probe | Vic-CAAAATATACAGCTATaGTAACC-MGBNFQ |
|  | Mut Probe | 6FAM-AAATATACAGCTATgGTAACC-MGBNFQ |
| *ZNRD1*_SNP2-G>A  (rs3869068) | Assay by Demand Assay (C__26544924_10) were ordered from Applied Biosystems (ABI, www.appliedbiosystem.com), primer/probe sequences are patented by ABI. | |
| *ZNRD1*_SNP3-A>C  (rs2074480) | Sense primer | 5’-GTTGTTTCAGCCACCTACTTTGC-3’ |
|  | Antisense primer | 5’-TCAGGTCCTTTTTGATGTCTGCATT-3’ |
|  | Wt Probe | Vic-TACTGCTCTaATGAAGC-MGBNFQ |
|  | Mut Probe | 6FAM-CTGCTCTcATGAAGC-MGBNFQ |
| *ZNRD1*_SNP4-T>G  (rs7758512) | Assay by Demand Assay (C___2437574_10) were ordered from Applied Biosystems (ABI, www.appliedbiosystem.com), primer/probe sequences are patented by ABI | |
| *ZNRD1*_SNP5-T>C  (rs9261129) | Assay by Demand Assay (C___2437551_10) were ordered from Applied Biosystems (ABI, www.appliedbiosystem.com), primer/probe sequences are patented by ABI. | |
| *ZNRD1*_SNP6-C>A  (rs2301753) | Assay by Demand Assay (C__15756685_20) were ordered from Applied Biosystems (ABI, www.appliedbiosystem.com), primer/probe sequences are patented by ABI | |
| *ZNRD1*_SNP7-T>C  (rs2074479) | Assay by Demand Assay (C__16164295_20) were ordered from Applied Biosystems (ABI, www.appliedbiosystem.com), primer/probe sequences are patented by ABI | |
| SNP | Primers/Probes | Oligonucleotide sequences |
| *HCP5*-T>G  (rs2395029) | Sense primer | 5’-TCTCACCCGCTGGTCTCT-3’ |
|  | Antisense primer | 5’-CAGGGTAGAAGGTCCTGGATTCT-3’ |
|  | Wt Probe | Vic-ACATTACAGCTGCCaCAGG-MGBNFQ |
|  | Mut Probe | 6FAM-CATTACAGCTGCCcCAGG-MGBNFQ |
| *HLA-C5’*-T>C  (rs9264942) | Assay by Demand Assay (C_29901957_10) were ordered from Applied Biosystems (ABI, www.appliedbiosystem.com), primer/probe sequences are patented by ABI | |
| *ZNRD1*-T>C  (rs9261174) | Sense primer | 5’-GCCAATACCTTGCTTGCCATTTT-3’ |
|  | Antisense primer | 5’-GCTGGAAGTATCACAGTACCTGACT-3’ |
|  | Wt Probe | Vic-CAAAATATACAGCTATaGTAACC-MGBNFQ |
|  | Mut Probe | 6FAM-AAATATACAGCTATgGTAACC-MGBNFQ |

Probe sequences in lower case represents different alleles. VIC and FAM, probe reporter fluorescent dyes. MGBNFQ, Molecular-Groove Binding Non-fluorescence Quencher hybridization probes which allow for using probes at lower melting temperature. Wt, wild type, Mut, mutated.

**Phenotypic endpoints**

The phenotypic endpoints were AIDS (1987 criteria) and the baseline CD4^+^ T cell count, steady-state viral load, nadir CD4^+^ T cell count, cumulative CD4^+^ T cell count and delayed type hypersensitivity (DTH) skin test reactivity as assessed and described previously [4,6].

***HLA* genotyping**

Genomic DNA was isolated from PBMCs by using QIAamp columns containing a silica membrane (Qiagen Inc, Valencia, CA) from the HIV-1 infected individuals recruited at Wilford Hall Medical Center, San Antonio, TX. *HLA* alleles were genotyped at the molecular level by using a combination of PCR-SSOP methods as described before [6]. Briefly, genomic DNA was amplified by using specific primers for *HLA-A* and *-B* provided by the manufacturer (Tepnel Lifecodes, Stamford, CT) to obtain products spanning exons 2-3 for class I alleles in a 100 µl PCR reaction. Five µl of the amplified product were run on the agarose gel to confirm the amplification and the remaining was applied to a series of positively charged nylon membranes (Amersham Hybond, Piscataway, NJ). After chemical denaturation with NaOH and neutralization, the membranes were hybridized with locus-specific probes labeled with alkaline phosphatase and the positive hybridization signal was revealed with Lumiphos 480 substrate (Tepnel Lifecodes) and a permanent radiographic record was obtained. Patterns of hybridization for allele assignment were by QuickType 2.0.51.

An additional degree of resolution was reached by using a fluorescent bead-based assay using the Luminex platform (Luminex, Austin, TX) for confirmation and enhanced discrimination of the HLA alleles. In brief, the LIFEMATCH_System (Tepnel Lifecodes) for *HLA* typing is based on the simultaneous detection of multicolored beads in suspension. In our study, one tube reaction containing the PCR amplified specific *HLA* product was hybridized with a set of probes attached to the fluorescent beads and further discrimination of positive hybridization was allowed by the use of Streptavidin-Phycoerythrin binding to PCR products carrying original biotin-labeled primers. *HLA* alleles were assigned by using the LIFEMATCH 2.2.1 program.

We further increased the resolution of *HLA-B*57* alleles in two groups: *HLA-B*5701* and *HLA-B*57* alleles that are not *B*5701*. *B*5701* allele was detected as described by Hammond et al [9]. Briefly, *B*5701* specific primers were used to selectively amplify *B*570101*, *B*570102*, and *B*570103* alleles and positive amplification was detected with SYBR green I (Invitrogen) in a 7900HT Sequence detection System. Human Growth Hormone primers were used as internal control for amplification. *HLA-B*5701* positive samples were determined by the presence of a fluorescence peak at 83.3 ^o^C in the melting curves after an additional dissociation step.

**Statistical methods**

Linkage disequilibrium among the HLA alleles and the studied polymorphisms was assessed using Lewontin’s D’ which was estimated using the Arlequin software package (version 3.0; 2005). Haplotypes were generated by the maximum likelihood estimation approach described by Stephens and Donnelly [10] using the PHASE software program. Survival analyses were conducted for time to AIDS (1987 criteria). We used Kaplan-Meier survival plots for depiction of the time-to-event curves and Cox proportional hazards models to estimate the RHs (with 95% CI) associated with the specific alleles/genotypes described herein. We tested the assumption of proportional hazards by plotting the Schoenfeld residuals and used the program stphtest (Stata 7.0, College Station, TX) to formally test the assumption. Schoenfeld residuals were calculated for each Cox proportional hazards model studied by using the Breslow-Peto approach. Mann-Whitney tests were used to compare differences of surrogate markers of disease progression between groups. Linear trend in proportions over categories of ordinal variable (for example, categories of time to AIDS and plasma viral load) was assessed using the Chi-square test for linear trend. Explained variation in the steady state viral loads was estimated from linear regression models and analysis of variance using the R^2^ statistic. Odds ratio for the possession of specific alleles classified on the basis of occurrence of an AIDS event and the time to its occurrence was estimated using multinomial logistic regression analysis.

**Supplementary Figures**

**Figure S1. Disease-influencing effects associated with *HLA-A10* status in HIV-positive EA subjects from the WHMC cohort who had not received HAART.** The data shown corresponds to that shown in the main text for Fig. 2B but are restricted to subjects who did not receive HAART. Data shows the Kaplan-Meier plots for HIV^+^ EA with or without *HLA-A10*, and the relative hazards (RH) and confidence intervals (CI) was estimated using Cox proportional hazards modeling.

**Figure S2. Association between *HLA-A10–ZNRD1* haplotypes and rates of HIV disease progression in the EA component of the WHMC cohort.** The *HLA-A10-ZNRD1* haplotypes were generated using the PHASE software and were named haplotypes (Hap) 1 to 5 as described in Figure 1C in the main text. For generating these haplotypes we used the genotyping information regarding *HLA-A10* status and the *ZNRD1* SNPs. As shown in Fig. 1C, haplotypes 1 and 4 are essentially similar except that haplotype 4 differs in the seventh SNP in or around the *ZNRD1-RNF39* locus. Additionally, the prevalence of haplotype 4 is significantly less than haplotype 1. Hence for statistical analyses we combined these two haplotypes together and designated them as haplotype 1+4 (Hap1+4). All other haplotypes are as defined in Figure 1C. Each panel represents Kaplan-Meier plots and the RH and 95% CI was estimated using Cox proportional hazards modeling. Within each panel different plots indicate the progression to AIDS (1987 criteria) for subjects who lacked that particular *A10-ZNRD1* haplotype (e.g., non-Hap2/non-Hap2), or who were heterozygous (e.g., Hap2/non-Hap2) or homozygous (e.g., Hap2/Hap2) for the haplotype. These results corroborate those shown in Figure 2A (middle panel), Figure 2B and 2D (main text) for the following reasons.

- First, homozygosity or heterozygosity for Hap1+4 was associated with a rapid rate of disease progression (panel A). This is consistent with the notion that haplotypes that lack *HLA-A10* in general are associated with a rapid rate of disease progression (panel A).
- Second, Hap2 bears the *ZNRD1-C* allele but lacks *HLA-A10* (Figure 1C, main text) and this haplotype is not associated with a protective effect in the heterozygous state (Hap2/non-Hap2), and in the homozygous state (i.e., Hap2/Hap2) it is associated with disease acceleration (panel B). Thus, this data is consistent with the results showing that heterozygosity for the *ZNRD1-C* alleles that lack *HLA-A10* (Hap2/non-Hap2) are not associated with disease-retardation and that homozygosity for the *ZNRD1-C* allele (Hap2/Hap2) is associated with disease acceleration (Figure 2D, main text; survival curves for genotypic groups 2 and 3).
- Third, Hap3 represents a haplotype that bears both *HLA-A10* and *ZNRD1-C* (Figure 1C), and those who are heterozygous for this haplotype (Hap3/non-Hap3) have a slower rate of disease progression compared to those lacking this haplotype (panel C). This is consistent with the survival curve shown for genotypic group 5 in the main text (Figure 2D).
- Fourth, Hap 5 represents a haplotype that bears *HLA-A10* but lacks *ZNRD1-C*, and heterozygosity for this haplotype (Hap5/non-Hap5) is associated with a slower rate of disease progression compared to those who lack this haplotype (non-Hap5/non-Hap5; panel D). This is consistent with the survival curve shown for genotypic group 4 in the main text (Figure 2D).

**Figure S3. Association between *HLA-C5’–HLA-B–HCP5* haplotypes and rates of HIV disease progression in the WHMC cohort.** The haplotypes were generated using the PHASE software and were named as described in Figure 1C (top) in the main text. Briefly, haplotype 1 is wild type for the *HLA-C5’* (*HLA-C5’-T*) and *HCP5* (*HCP5-T*) SNPs and lacks the *HLA-B*57* allele; Haplotype 2 represents a mutated *HLA-C5’* SNP (*HLA-C5’-C*) on the haplotypic background of a wild-type *HCP5* SNP and absence of *HLA-B*57* alleles; Haplotype 3 represents that haplotype that contains the mutation in both the SNPs (*HLA-C5’-C* and *HCP5-G*) on the haplotypic background of the *HLA-B*57* allele; and Haplotype 4 represents the haplotype which contains a *HLA-B*57* allele but wild-type states for both the SNPs. Each row in the figure represents the disease-influencing effects associated with each of these four haplotypes. Within each row there are three panels – the left-most panel is for all subjects in the cohort, the middle panel is for the EA component while the right-most panel is for the AA component of the HIV^+^ WHMC cohort. Each panel represents Kaplan-Meier plots and the relative hazards (RH) and 95% CI estimated using Cox proportional hazards modeling. Within each panel different plots indicate the progression to AIDS for subjects who lacked that particular haplotype, who were heterozygous for that haplotype and those who were homozygous for that haplotype. These results corroborate those shown in Figure 3, A to C in the main text as they show the following.

1. Panel A shows that homozygosity but not heterozygosity for haplotype 1 is associated with a rapid rate of disease progression in the entire cohort and the EA component of the cohort. Although showing a similar trend in AA, these associations do not achieve significance. These findings suggest that as haplotype 1 lacks protective polymorphisms/alleles such as *HLA-C5’-C* and/or *B*57* it may be associated with a faster rate of disease progression.
2. Panel B shows that heterozygosity and homozygosity for haplotype 2 is associated with a reduced rate of disease progression which is evident mainly in the EA component of the cohort. As haplotype 2 contains the *HLA-C5’-C* allele but lacks *HLA-B*57* or *HCP5-G* alleles, these findings indicate that the *HLA-C5’-C* allele in itself is associated with a reduced rate of disease progression.
3. Panel C shows that a haplotype that contains *HLA-C5’-C*, *HLA-B*57* and *HCP5-G* (haplotype 3) is associated with a trend for disease protection in EA.
4. Panel D shows that a haplotype that lacks both *HLA-C5’-C* and *HCP5-G* alleles but contains *HLA-B*57* is associated with a significantly slower rate of disease course in both EA and AA.

**Figure S4. Disease-influencing effects associated with *HLA-B*57-NPD* and HLA-*B*57-PD* genotypes in subjects who had not received HAART.** The data shown corresponds to that shown in the main text for Figure 4D but are restricted to subjects who did not receive HAART. Data shows the Kaplan-Meier plots for subjects categorized to the indicated genotypes. The relative hazards (RH) and confidence intervals (CI) was estimated using Cox proportional hazards modeling.

**References**

1. Gonzalez E, Bamshad M, Sato N, Mummidi S, Dhanda R, et al. (1999) Race-specific HIV-1 disease-modifying effects associated with CCR5 haplotypes. Proc Natl Acad Sci U S A 96: 12004-12009.

2. Gonzalez E, Dhanda R, Bamshad M, Mummidi S, Geevarghese R, et al. (2001) Global survey of genetic variation in CCR5, RANTES, and MIP-1alpha: impact on the epidemiology of the HIV-1 pandemic. Proc Natl Acad Sci U S A 98: 5199-5204.

3. Gonzalez E, Rovin BH, Sen L, Cooke G, Dhanda R, et al. (2002) HIV-1 infection and AIDS dementia are influenced by a mutant MCP-1 allele linked to increased monocyte infiltration of tissues and MCP-1 levels. Proc Natl Acad Sci U S A 99: 13795-13800.

4. Gonzalez E, Kulkarni H, Bolivar H, Mangano A, Sanchez R, et al. (2005) The influence of CCL3L1 gene-containing segmental duplications on HIV-1/AIDS susceptibility. Science 307: 1434-1440.

5. Dolan MJ, Kulkarni H, Camargo JF, He W, Smith A, et al. (2007) CCL3L1 and CCR5 influence cell-mediated immunity and affect HIV-AIDS pathogenesis via viral entry-independent mechanisms. Nat Immunol 8: 1324-1336.

6. Ahuja SK, Kulkarni H, Catano G, Agan BK, Camargo JF, et al. (2008) CCL3L1-CCR5 genotype influences durability of immune recovery during antiretroviral therapy of HIV-1-infected individuals. Nat Med 14: 413-420.

7. Catano G, Agan BK, Kulkarni H, Telles V, Marconi VC, et al. (2008) Independent effects of genetic variations in mannose-binding lectin influence the course of HIV disease: the advantage of heterozygosity for coding mutations. J Infect Dis 198: 72-80.

8. Burt TD, Agan BK, Marconi VC, He W, Kulkarni H, et al. (2008) Apolipoprotein (apo) E4 enhances HIV-1 cell entry in vitro, and the APOE epsilon4/epsilon4 genotype accelerates HIV disease progression. Proc Natl Acad Sci U S A 105: 8718-8723.

9. Hammond E, Mamotte C, Nolan D, Mallal S (2007) HLA-B*5701 typing: evaluation of an allele-specific polymerase chain reaction melting assay. Tissue Antigens 70: 58-61.

10. Stephens M, Smith NJ, Donnelly P (2001) A new statistical method for haplotype reconstruction from population data. Am J Hum Genet 68: 978-989.

**Table S1. Distribution of alleles that categorize to the *HLA-A10* serogroup among European Americans (EA) and African Americans (AA) in the WHMC HIV^+^ cohort.**

Number of individuals and percentage of subjects bearing an allele that categorizes to the *HLA-A10* serogroup

| Allele | Race | |
| --- | --- | --- |
|  | EA | AA |
| *A*25* | 33 (52.4%) | 1 (1.2%) |
| *A*26* | 23 (36.5%) | 16 (20.7%) |
| *A*34* | 3 (4.8%) | 31 (40.2%) |
| *A*66* | 4 (6.3%) | 29 (37.6%) |
| Total | 63 | 77 |

Percentage of alleles that categorize to the *A-10* serogroup among all HIV positive subjects according to race/ethnicity

| Allele | Race/Ethnicity | |
| --- | --- | --- |
|  | EA | AA |
| *A*25* | 4.97 | 0.22 |
| *A*26* | 3.46 | 3.54 |
| *A*34* | 0.44 | 6.87 |
| *A*66* | 0.60 | 6.43 |
| Total | 9.47 | 17.1 |

**Table S2. Distribution of *HLA-B*57, -B*5701* and *HCP5-G* alleles in HIV-positive subjects of the WHMC cohort.**

| *HLA-B*57/B*5701* allele | *HCP5-G* allele | All | |  | EA | |  | AA | |
| --- | --- | --- | --- | --- | --- | --- | --- | --- | --- |
|  |  | n | % |  | n | % |  | n | % |
| *HLA-B*57*+ | + | 46 | 4.1 |  | 40 | 6.2 |  | 6 | 1.3 |
|  | − | 41 | 3.6 |  | 5 | 0.8 |  | 33 | 7.4 |
| *HLA-B*57*− | + | 4 | 0.3 |  | 2 | 0.3 |  | 2 | 0.4 |
|  | − | 1035 | 92 |  | 603 | 92.7 |  | 406 | 90.8 |
| *HLA-B*5701*+ | + | 46 | 4.1 |  | 40 | 6.2 |  | 6 | 1.3 |
|  | − | 4 | 0.3 |  | 2 | 0.3 |  | 2 | 0.4 |
| *HLA-B*5701*− | + | 4 | 0.3 |  | 2 | 0.3 |  | 2 | 0.4 |
|  | − | 1072 | 95.2 |  | 606 | 93.2 |  | 437 | 97.8 |

+, present; −, absent; All, all subjects; EA, European American; AA, African American

**Table S3: Distribution of the SNPs in the WHMC cohort and association between these SNPs and risk of acquiring HIV-1**

| SNP | Genotype | HIV+ | HIV- | OR | 95% CI | *P* |
| --- | --- | --- | --- | --- | --- | --- |
| *European and Hispanic Americans* | | | | | | |
|  | | | | | | |
| *ZNRD1-T>C*  (rs9261174) | TT | 583 | 465 | 1.00 |  |  |
|  | TC | 160 | 141 | 0.90 | 0.70 – 1.18 | 0.4470 |
|  | CC | 10 | 16 | 0.50 | 0.22 – 1.11 | 0.0821 |
|  | H-W E *P* | 0.7932 | 0.1836 |  |  |  |
|  |  |  |  |  |  |  |
| *HLA-C5’*-*T>C*  (rs9264942) | TT | 314 | 252 | 1.00 |  |  |
|  | TC | 347 | 290 | 0.96 | 0.76 – 1.20 | 0.7271 |
|  | CC | 92 | 80 | 0.93 | 0.65 – 1.30 | 0.6461 |
|  | H-W E *P* | 0.7968 | 0.8092 |  |  |  |
|  |  |  |  |  |  |  |
| *HCP5-T>G*  (rs2395029) | TT | 709 | 582 | 1.00 |  |  |
|  | TG | 44 | 40 | 0.90 | 0.58 – 1.41 | 0.6507 |
|  | GG | 0 | 0 | --- | --- | --- |
|  | H-W E *P* | 0.4089 | 0.4073 |  |  |  |
| *African Americans* | | | | | | |
|  | | | | | | |
| *ZNRD1-T>C*  (rs9261174) | TT | 261 | 222 | 1.00 |  |  |
|  | TC | 170 | 150 | 0.96 | 0.72 – 1.28 | 0.7996 |
|  | CC | 39 | 35 | 0.94 | 0.58 – 1.54 | 0.8302 |
|  | H-W-E *P* | 0.1355 | 0.1853 |  |  |  |
|  |  |  |  |  |  |  |
| *HLA-C5’-T>C*  (rs9264942) | TT | 242 | 212 | 1.00 |  |  |
|  | TC | 187 | 161 | 1.02 | 0.77 – 1.35 | 0.9033 |
|  | CC | 41 | 34 | 1.05 | 0.65 – 1.72 | 0.8265 |
|  | H-W-E *P* | 0.5709 | 0.1921 |  |  |  |
|  |  |  |  |  |  |  |
| *HCP5-T>G*  (rs2395029) | TT | 462 | 400 | 1.00 |  |  |
|  | TG | 8 | 7 | 0.99 | 0.36 – 2.58 | 0.9838 |
|  | GG | 0 | 0 | --- | --- | --- |
|  | H-W-E *P* | 0.8524 | 0.0811 |  |  |  |
|  |  |  |  |  |  |  |

OR, odds ratios; CI, confidence interval. H-W E, Hardy-Weinberg Equilibrium
