# Supplementary figures and images for: HIV-1 Disease-Influencing Effects Associated with ZNRD1, HCP5 and HLA-C Alleles Are Attributable Mainly to Either HLA-A10 or HLA-B*57 Alleles
Source: PLoS One. 2008 Nov 4;3(11):e3636. doi: 10.1371/journal.pone.0003636 (PMC2574440; doi:10.1371/journal.pone.0003636)

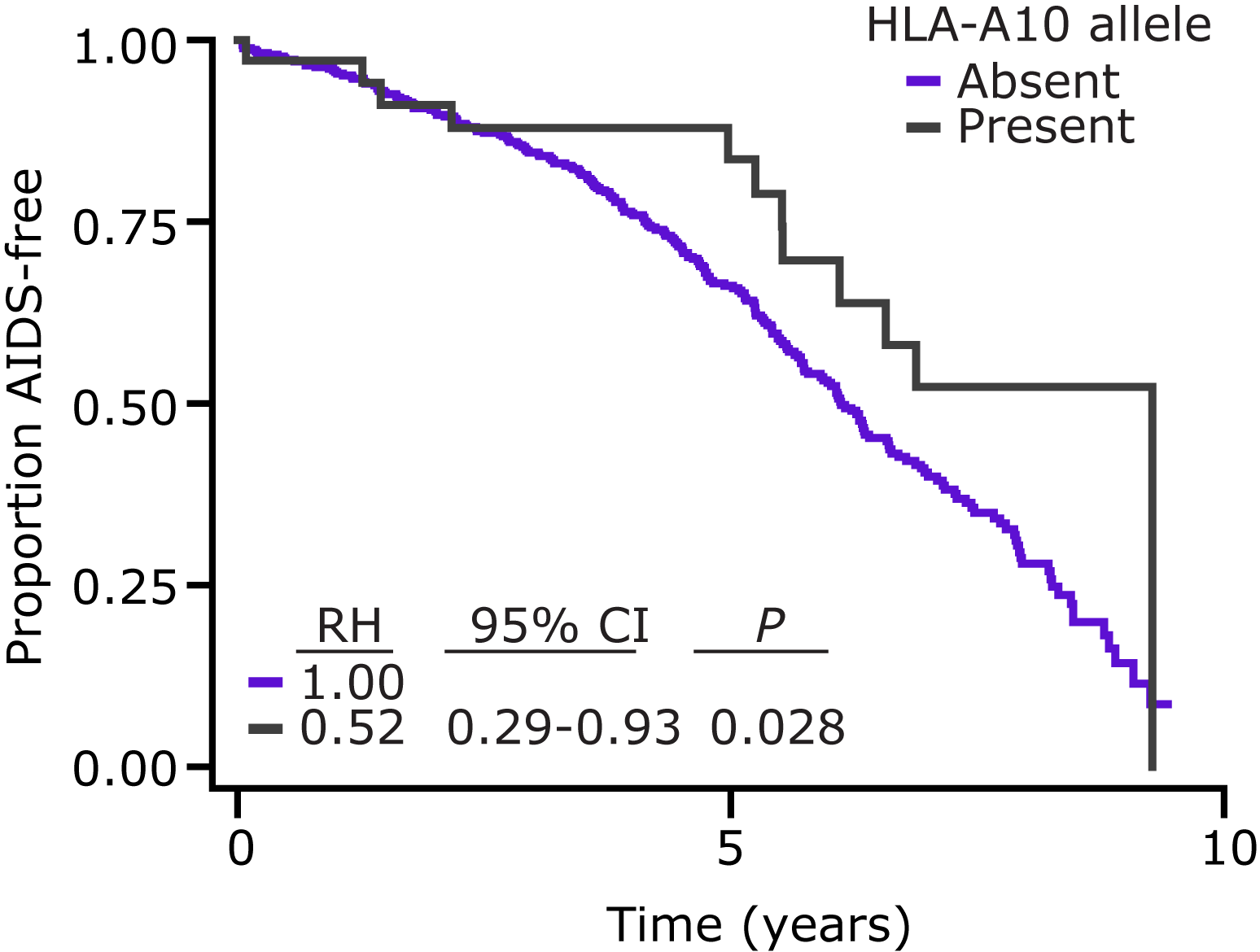

Supplement: Figure S1 — Disease-influencing effects associated with HLA-A10 status in HIV-positive EA subjects from the WHMC cohort who had not received HAART. (0.26 MB TIF) [file pone.0003636.s002.tif]

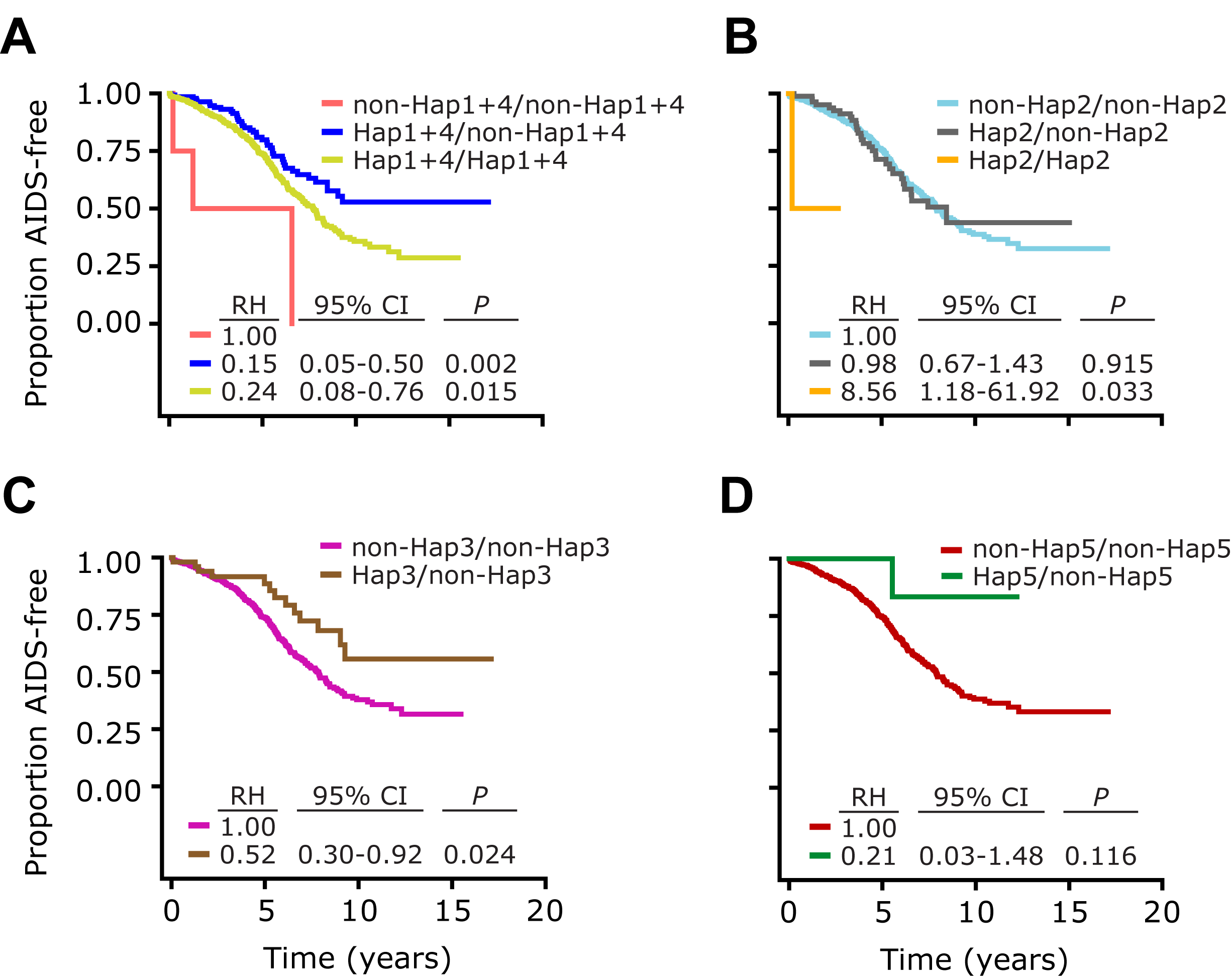

Supplement: Figure S2 — Association between HLA-A10-ZNRD1 haplotypes and rates of HIV disease progression in the EA component of the WHMC cohort. (0.58 MB TIF) [file pone.0003636.s003.tif]

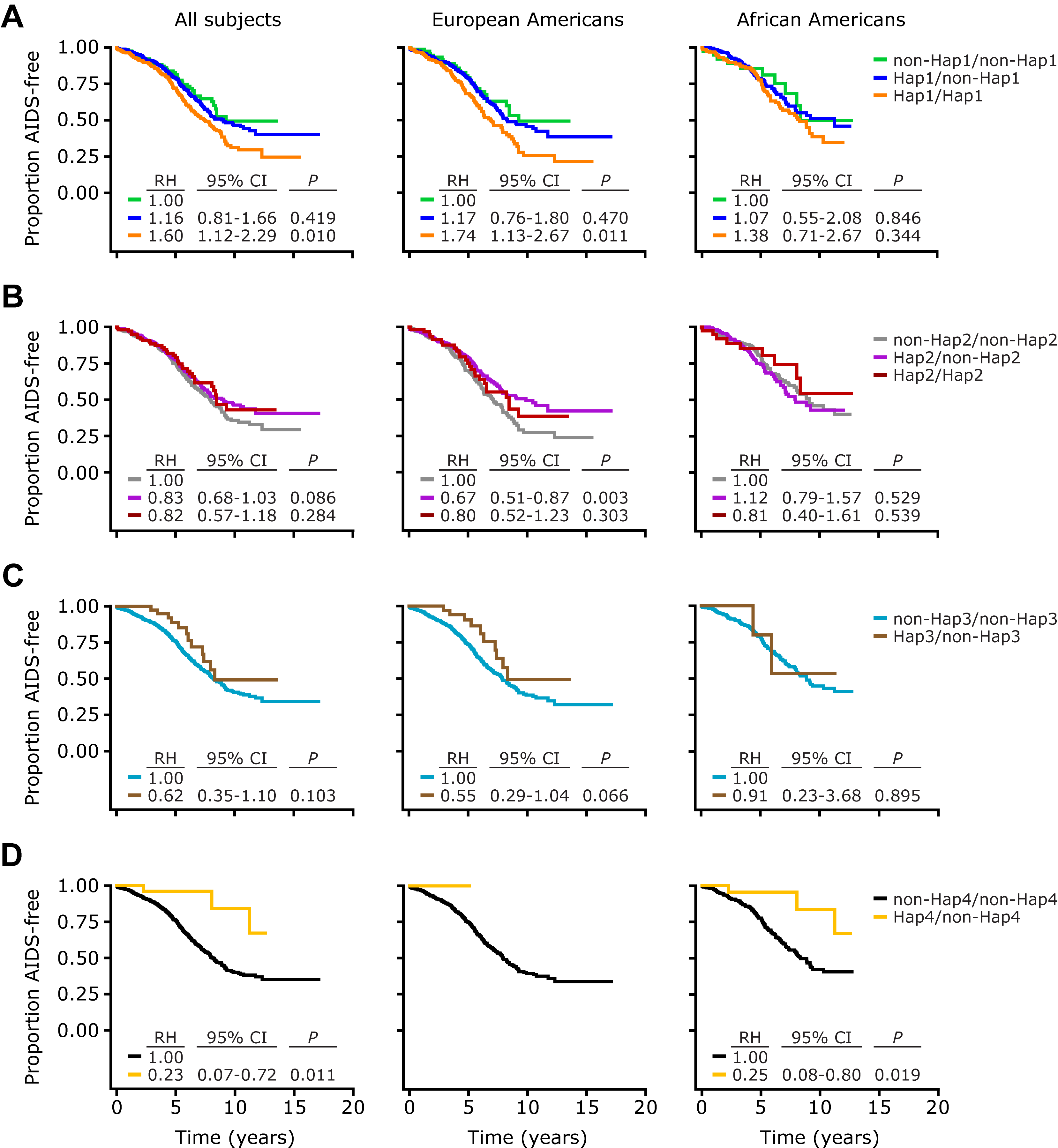

Supplement: Figure S3 — Association between HLA-C5′-HLA-B-HCP5 haplotypes and rates of HIV disease progression in the WHMC cohort. (1.06 MB TIF) [file pone.0003636.s004.tif]

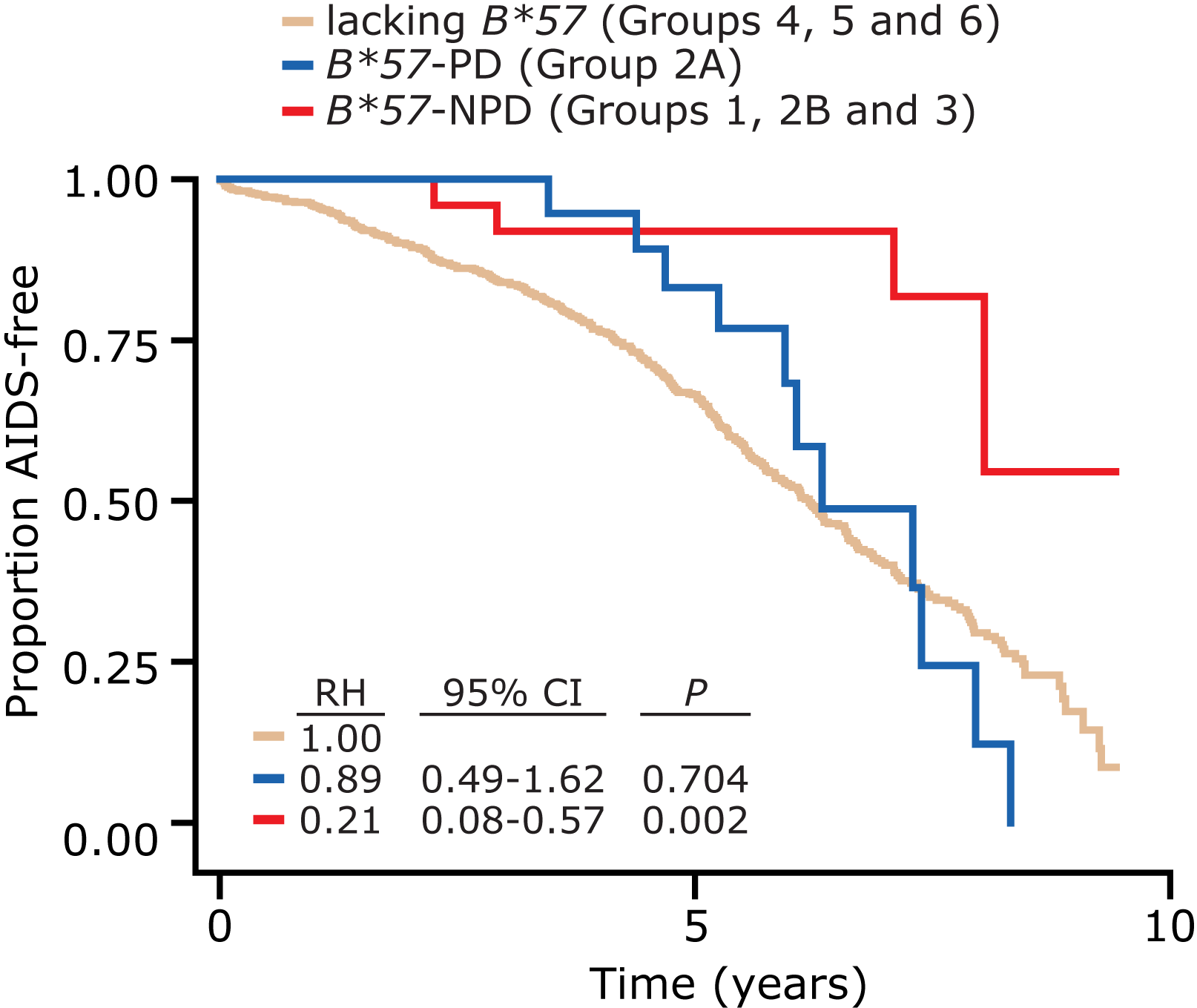

Supplement: Figure S4 — Disease-influencing effects associated with HLA-B*57-NPD and HLA-B*57-PD genotypes in subjects who had not received HAART. (0.33 MB TIF) [file pone.0003636.s005.tif]
